# Experimental evolution of *Plasmodium yoelii* in single and helminth-coinfected mice

**DOI:** 10.1186/s12936-025-05764-1

**Published:** 2025-12-26

**Authors:** Aloïs Dusuel, Luc Bourbon, Emma Groetz, Mickaël Rialland, Benjamin Roche, Bruno Faivre, Gabriele Sorci

**Affiliations:** 1 CTM - Center for Translational and Molecular Medicine, INSERM UMR 1231, Université Bourgogne Europe, 21000 Dijon, France; 2 LabEx LipSTIC, 21000 Dijon, France; 3 Biogéosciences, CNRS UMR 6282, Université Bourgogne Europe, 6 Boulevard Gabriel, 21000 Dijon, France; 4 MIVEGEC, IRD, CNRS, Université de Montpellier, 34090 Montpellier, France

**Keywords:** Coinfection, *Heligmosomoides polygyrus*, Mismatched environments, Nematodes, Parasite adaptation, *Plasmodium yoelii*, Virulence

## Abstract

**Background:**

Coinfection has the potential to affect key traits describing the infection dynamics, the severity of the disease and *in fine* parasite fitness. However, despite its pervasiveness, experimental work investigating how parasites adapt to the conditions provided by a coinfected host is mostly missing.

**Methods:**

We adopted an experimental evolution approach to investigate if coinfection with the nematode *Heligmosomoides polygyrus* (Hp) affected the infection dynamics and virulence of the murine malaria parasite *Plasmodium yoelii* (Py). To this purpose, lines of Py were passaged either in single infected hosts (SI-lines) or in hosts that had been previously infected with Hp (COI-lines). After five and seven passages, the infection dynamics and virulence of evolved lines were compared to the ancestral Py population during single infection trials. COI-lines were also used to infect hosts during coinfection trials, allowing us to compare within-host Py replication when the environment during the evaluation trials matched the environment experienced during the passages and when the two environments were mismatched.

**Results:**

We found that serial passages increased parasitemia and Py virulence, due to the competitive advantage of genotypes with the fastest replication rate, but SI-lines and COI-lines had relatively similar replication rate and virulence. Hosts infected with evolved lines of Py were also less tolerant (steeper slope between red blood cell counts and parasitemia) but there was no difference between SI-lines and COI-lines. Finally, we found that when COI-lines were used during single infection trials (mismatched environments), they had a slower early replication rate compared to matched-environment trials.

**Conclusions:**

We did not find strong evidence supporting a divergence between the virulence of SI-lines and COI-lines, possibly due to the cost of virulence paid by COI-lines. However, Py rapidly adapted to the environmental conditions provided by single infected or coinfected hosts, as shown by the slower replication rate found in mismatched-environment trials.

**Supplementary Information:**

The online version contains supplementary material available at 10.1186/s12936-025-05764-1.

## Background

Why parasites kill their hosts has puzzled biologists for decades [67]. Indeed, parasites (general term including both micro and macroparasites) usually rely on host survival to produce propagules, and host death inevitably incurs the end of the infectious period (the period during which parasites produce transmissible stages). Based on this simple idea, it was believed that parasite virulence (the degree of infection-induced damage potentially leading to host death) was a maladaptive trait and that selection should consistently favor more benign parasite strains (Smith’s law of declining virulence, [46]). This view was nevertheless at odds with the observation that some parasites have maintained high levels of virulence over time. A solution to the puzzle was finally provided by the seminal work of [2–4], who suggested that parasite virulence can evolve up and down depending on the balance between the benefits arising from increased host exploitation (i.e., increased within-host replication rate) and the costs arising from host death (i.e., decreased infectious period). Since then, this trade-off model of virulence evolution has attracted considerable attention both from theoreticians and empiricists who have investigated the assumptions of the model and the generality of the predictions (e.g., [10, 14, 26, 66]).

Whatever the direction of the selection on virulence, parasites are expected to adapt to the prevailing environmental conditions, as to maximize transmission success (e.g., [42, 52]). Experimental evolution in the lab has consistently shown that parasites passaged in a novel host become adapted to it while becoming maladapted to the ancestral host [22, 51]. Evidence of parasite adaptation to novel hosts comes also from natural outbreaks. For instance, during the Ebola virus outbreak in west Africa, the glycoproteins allowing the viral entrance in the host cells increased their affinity towards the human receptor while losing affinity towards the ancestral bat receptor [61], indicating ongoing viral adaptation to the human host.

In natural populations, hosts are exposed to a multitude of parasites that can concomitantly infect the same individuals [62]. Coinfection has the potential to deeply change the environment encountered by parasites compared to single infected hosts for several reasons [17, 23, 25, 27, 28, 32, 49, 55]. Indeed, depending on the nature of the coinfecting parasites, the adaptive response of the host can be polarized towards different immune effectors (Th1/Th17 vs. Th2), making the ground more or less favorable for host exploitation [41, 56]. In addition to this, parasites (e.g., helminths) can also directly interfere with the host immune response and the associated immune modulation can promote the infection by competing parasites [29, 53]. Finally, coinfecting parasites might directly compete for resources provided by the host, through bottom-up regulatory mechanisms [7].

Malaria is still one of the deadliest infectious diseases worldwide, with an estimated number of about 600,000 deaths in 2023 [65]. Malaria endemicity largely overlaps with the zones where helminthiases (soil-transmitted helminth infections) are also highly prevalent. As a consequence, coinfection between malaria and helminths is common among the poorest populations living in the intertropical area [1, 35]. Several epidemiological studies have investigated the effect of the coinfection on the severity of malaria symptoms with somehow mixed results since both aggravating and protective effects have been reported [15, 31, 44]. Although soil-transmitted helminths do not exert the same mortality burden as malaria, they nevertheless represent a public health concern due to the high prevalence of infection and their effect on infant physical and cognitive development. Massive deworming is one of the control strategies that is regularly implemented to reduce the burden of infection with soil-transmitted helminths [64], but its efficacy and rationale has been debated [30, 58], on the ground, among others, of the risk of emergence of drug-resistant strains [11, 60]. Moreover, another aspect linked to deworming that has been barely considered is the possible feedback on coinfecting microparasites (e.g., malaria) and their adaptive response to the clearance of helminth infection. In one of these few attempts, Fenton [24] built a model where he analyzed the possible outcomes of deworming on two key traits of a hypothetical coinfecting microparasite, the basic reproduction number and virulence. The model explicitly considered different types of within-host interactions between worms and pathogens, where for instance worms aggravate the severity of the infection with the microparasite, or improve host recovery rate. The findings suggested that the outcome of deworming is largely context-dependent, according to the type of within-host interaction. In particular, when helminths aggravate the severity of the pathogen infection (increase in host mortality), deworming should select for increased pathogen virulence. Similarly, when helminths weaken the host recovery rate, the model predicted that deworming should promote pathogen virulence, at least when the natural worm burdens are low to moderate.

Here, we wished to investigate if malaria parasites can adapt to the conditions provided by a coinfected host using a model involving two murine parasites, *Plasmodium yoelii* (hereafter Py) and the gastrointestinal nematode *Heligmosomoides polygyrus* (hereafter Hp). In a recent paper, we showed that Py incurs higher costs when infecting hosts already infected with Hp (compared to the single infection) [21]. We also showed that the changes in the host immune response induced by Hp promoted Py replication and slowed down host recovery rate [21]. Therefore, when mosquitoes bite and transmit Py to a host already harboring a Hp infection, Py will likely experience a very distinct immune environment compared to the one provided by a non-infected host. Here, we adopted an experimental evolution approach where Py was passaged either in single infected (potentially reproducing the environment provided by a dewormed host) or in coinfected (with Hp infecting first) hosts, in a one-sided evolution design. After five and seven passages, the evolved Py lines were used to infect mice in single infection trials and the symptoms elicited by the infection were compared to those elicited by the ancestral Py population. We measured several proxies of disease severity, including traits related to the host capacity to tolerate the cost of the infection. Moreover, Py lines evolved in coinfected hosts were also used in coinfection trials. This allowed us to investigate whether lines passaged and tested in the same environmental conditions (matched environments) fared better than lines experiencing mismatched environments. This is supposed to reproduce the conditions encountered by *Plasmodium* potentially switching from wormed to dewormed hosts.

## Methods

### Experimental animals

C57BL/6JRj female mice (7–10 weeks old) were purchased from Janvier Labs (Le Genest-Saint-Isle, France), housed in cages containing 5 individuals under pathogen-free conditions, and maintained under a constant temperature of 24 °C and a photoperiod of 12 h:12 h light:dark with *ad libitum* access to water and standard chow diet (A03-10, Safe, Augny, France). All mice were acclimatized to the housing conditions during, at least, 7 days prior to the start of the experiments, were monitored twice a day to check health status, and euthanized by cervical dislocation under anesthesia with isoflurane either if they reached previously defined end points (clinical scores based on weight loss, behavior and physical appearance, and considered as mortality events in the survival analysis) or at day 3 and 14 post-infection (p..i.) for terminal collection of blood and spleen (and considered as censored events in the survival analysis).

### Serial passages

At the beginning of the experiment, one group of five mice was infected with *Plasmodium yoelii* 17XNL by intra-peritoneal (i.p.) injection with 5 × 10^5^ infected red blood cells (iRBC) suspended in 0.1 ml of PBS (single infection group), and another group of five mice was first infected with *Heligmosomoides polygyrus bakeri* by oral gavage with L3 larvae (350 larvae suspended in 0.2 ml of drinking water) and after 28 days infected with Py as described above (coinfection group). Infected RBC used for single infections and coinfections came from the same stock.

**Fig. 1:**
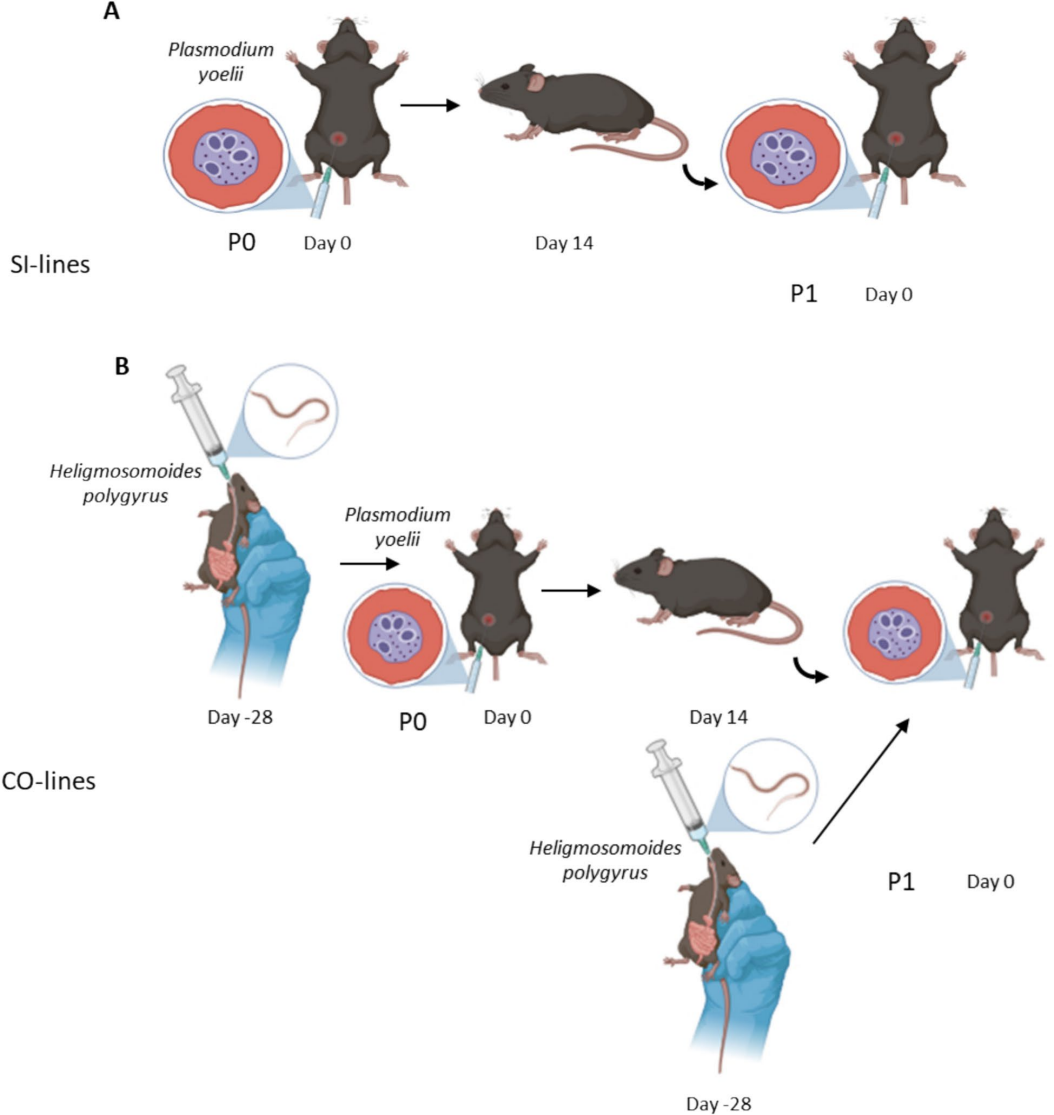
Schematic view of the experimental evolution setup. Py was serially passaged in single infected (SI-lines) or coinfected hosts (COI-lines). In the single infection treatment, mice were infected with Py (i.p. injection) at day 0 and Py was transmitted to a new host at day 14 p.i. (corresponding to day 0 for this new host). In the coinfection treatment, hosts were infected with Hp at day − 28, infected with Py at day 0, and Py was transmitted to a new host at day 14 p.i. (corresponding to day 0 for this new host). Five mice were infected per passage, with one mouse providing Py to infect a single new host, corresponding to five replicated lines. Only Py was passaged, Hp coming from the stock population (i.e., only Py was allowed to evolve). After 5 and 7 passages (and 10 passages for the SI-lines only) blood was collected and deep frozen for subsequent use during the evaluation trials of the selected lines. Figure created in BioRender

At day 14 post Py infection, Py from the single infection group was passaged to a new batch of five mice that received it as a single infection (5 × 10^5^ iRBC), and Py from the coinfection group was passaged to a new batch of five mice that had been previously infected with Hp (28 days prior to the Py infection). Therefore, we had two treatments: either Py was passaged in single infected mice (SI-lines) either in coinfected mice (COI-lines). Since Py was transmitted from one mouse to a new mouse, each treatment had 5 replicated Py lines. Infection with Hp was done using the stock population of nematodes; in other words, only Py was allowed to respond to the treatment over the passages, in a one-sided evolution design. This setup was repeated up to passage 10 for the SI-lines, and up to passage 7 for the COI-lines, due to the mortality during the passages for this treatment. At passage 5, 7 and 10, we proceeded with the terminal collection of peripheral blood for each of the five mice from each treatment. Blood was centrifuged (7 min, 3000 rpm, 4 °C), plasma was discarded and replaced by an equivalent volume of a glycerol freezing solution (100 ml of 0.9% NaCl solution + 4.2 g of sorbitol + 39 ml of glycerol). Tubes were then gently mixed and stored in liquid nitrogen until use for the evaluation trials. Figure 1 schematically illustrates the experimental design used for the passages.

### Evaluation trials

The SI-lines and the COI-lines were evaluated after passage 5 and 7 (and 10 for the SI-lines alone). To this purpose, Py was first revitalized. Blood tubes were removed from the liquid nitrogen, thawed to 37 °C and 100 µl of the suspension were injected (i.p.) to a naïve mouse. One mouse was used to revitalize one Py line per passage and treatment. At day 14 p.i., we collected 10 µl of blood to count RBC, and to make a blood smear to assess parasitemia. This allowed us to compute the volume of blood needed to infect the mice used for the evaluation trials with the dose of 5 × 10^5^ iRBC. One line was lost during this revitalization phase (one of the SI-lines after passage 7) because Py failed to replicate. Fifteen mice were used for the evaluation trials (single infection trials) per passage/treatment. At passage 7, the evaluation trials were repeated using another batch of 15 mice (the total number of mice used for the evaluation trials after passage 7 was therefore 30 per treatment). The health status and parasitemia of mice used for the evaluation trials were monitored during 14 days post Py infection. This set up allowed us to compare the infection dynamics and virulence of evolved SI-lines and COI-lines in single infected hosts compared to the ancestral Py population, for which the infection dynamics and virulence were previously described and published [21].

For the COI-lines at passage 7, we also performed evaluation trials in coinfected hosts (mice that had been previously infected with Hp at day − 28). Thirty mice were used for these coinfection trials, using two batches of 15 mice, and the infection dynamics and virulence assessed as for the single infection trials. Therefore, we could compare the performance of COI-lines when encountering the same environmental conditions as those experienced during the passages (matched environments) to the performance of Py encountering mismatched environments, which reproduces the case when coinfected hosts are dewormed.

### Monitoring of health status, hematological and plasma parameters, and Py parasitemia

Mice were weighed at day 0, 3, 7, 9 and 14 post Py infection (± 0.1 g) and body mass was used as an integrative marker of host health. RBC were counted at day 0, 3, 9, 14 post Py infection using a SCIL Vet abc Plus + hematology analyzer (Horiba Medical, Montpellier, France) on 10 µl of blood collected from the tail tip and disposed in 0.5 ml tubes with 1 µl of heparin (Héparine Choay^®^ 25 000 UI/5 ml, Sanofi-Aventis, Gentilly, France). Parasitemia (proportion of iRBC) was assessed at day 3, 7, 9, and 14 post Py infection by smearing a drop of blood on a slide. Slides were fixed with methanol and then stained with a Giemsa RS solution (Carlo Erba Reagents, Val de Reuil, France) (10% v/v in phosphate buffer) for 15 min and rinsed with water. Infected RBC were counted using an optical microscope (Eclipse E600, Nikon Corporation, Tokyo, Japan) under magnification × 1000.

At day 3 and 14 post Py infection, five and ten mice per treatment (unless they had reached the end point prior to day 14 p.i.), respectively, were euthanized by cervical dislocation under isoflurane anesthesia, for terminal collection of total peripheral blood and spleen. Plasma was separated from total blood by centrifugation (7 min 3000 rpm 4 °C), aliquoted and stored at − 80 °C. Spleen was removed, about one third was cut and immediately frozen in liquid nitrogen for RT-qPCR, and the remaining was kept on ice in PBS before the flow cytometry staining procedure.

Plasma levels of mouse erythropoietin (EPO) and heme oxygenase-1 (HO-1) were assessed in duplicates with EM28RB ELISA kit from Invitrogen^™^ (Carlsbad, California, USA) and ab229431 ELISA kit from Abcam (Cambridge, UK), respectively. Plates were read with a SpectraMax iD3 multi-mode microplate reader (Molecular Devices, San Jose, California, USA).

### RNA extraction and qPCR for IFN-γ and IL-10 gene expression

Spleen tissue was homogenized in TRIzol^™^ Reagent (Invitrogen^™^, Carlsbad, California, USA) under strong agitation using 0.5 mm glass beads and a Precellys^®^ 24 Touch homogenizer (Bertin Technologies, Montigny-le-Bretonneux, France). RNA extraction was performed following the manufacturer’s instructions. RNA concentration was measured with a NanoPhotometer^®^ N50 (Implen, Munich, Germany). Reverse transcription was performed with a High-Capacity cDNA Reverse Transcription Kit (Applied Biosystems^™^, Foster City, California, USA) from 1.5 µg total RNA. Quantitative PCR was performed in duplicates with PowerUp^™^ SYBR^™^ Green Master Mix (Applied Biosystems^™^) on a QuantStudio^™^ 3 Real-Time PCR System (Applied Biosystems^™^). We used two housekeeping genes (β-actin and GAPDH); β-actin provided more consistent values (less intra-group variability) and therefore we used it as the reference gene. Primer sequences are reported in the supplementary material (table S1), and were checked for specificity using the Primer-BLAST tool (NCBI). Melt curve analysis was also performed in all samples to check for the presence of nonspecific amplification products.

### Assessment of FoxP3^+^ Treg cells

Spleens were homogenized with a 70 µm cell strainer and washed two times with PBS to obtain a single-cell suspension. RBC were removed from suspensions with eBioscience^™^ RBC Lysis Buffer (Invitrogen^™^) (3 min incubation at RT) and washed two times with PBS before staining procedure.

Live cells were counted with viability trypan blue dye and dispatched in 96-well V bottom plates to obtain 1.10^7^ cells/well. Cells were then stained with LIVE/DEAD^™^ Fixable Blue Dead Cell Stain Kit (Invitrogen^™^) in PBS for 30 min at 4 °C, incubated in stain buffer with BD Fc Block^™^ (BD Pharmingen^™^, Franklin Lakes, New Jersey, USA) for 15 min at RT (0.5 µg/well), and stained with surface antibodies in brilliant stain buffer (see table S2 for details on the markers used) for 30 min at 4 °C. Cells were then fixed with eBioscience^™^ Foxp3/Transcription Factor Staining Buffer Set (Invitrogen^™^) for 40 min at RT prior permeabilization and intracellular staining in brilliant stain buffer (table S2) for 30 min at RT. Cells were resuspended in stain buffer, filtered with 100 µm nylon mesh and analyzed on a 4-laser Cytek Aurora^™^ spectral flow cytometer (Cytek^®^ Biosciences, Fremont, California, USA) in the ImaFlow facility part of the US58 BioSanD (Dijon, France). Events were gated and analyzed with SpectroFlo^®^ v3.0.0 software (Cytek^®^ Biosciences) (see figure S1 for the gating strategy).

### Statistical analyses

The aim of this work was to compare whether experimental evolution of Py in single infected or in coinfected hosts produced a divergence with respect to the ancestral population. The data for the ancestral population were part of a previous experiment [21], therefore, we expressed the phenotypic traits of the evolved lines relative to the ancestral population as log-ratios, which allows to easily visualize whether the experiment resulted in a divergence (up = above zero or down = below zero) from the initial values. Log-ratios were computed as the difference in ln-transformed values:

1 $${\mathrm{logratio}} = \ln \left( {y_{{{\mathrm{evolved}}}} } \right) - \ln \left( {mean_{{{\mathrm{ancetral}}}} } \right)$$

We also computed the variance of the log-ratios as the sum of the variance of ln(*y*_*evolved*_) and the variance of ln(*mean*_*ancestral*_), (the covariance between *y*_*evolved*_ and *mean*_*ancestral*_ being nil), and used it to compute weights:

2 $$w_{logratio} = \frac{1}{{Var_{logratio} }}$$

that were included in the LMM (see below). This allowed us to deal with the uncertainty around the mean values of the ancestral population (additive variance making the propagation of uncertainty trivial, [8]). When dealing with proportions (parasitemia and the proportion of FoxP3^+^ cells within CD4^+^ T lymphocytes), we computed logit-ratios as:

3 $$logitratio = {\mathrm{ln}}\left( {\frac{{y_{evolved} }}{{1 - y_{evolved} }}} \right) - {\mathrm{ln}}\left( {\frac{{mean_{ancestral} }}{{1 - mean_{ancestral} }}} \right)$$

For the gene expression of IFN-γ and IL-10, we computed:

4 $${\Delta\Delta\text{ Ct}} = {\Delta\text{ Ct}}_{{{\mathrm{evolved}}}} - {\Delta\text{ Ct}}_{{{\mathrm{ancestral}}}}$$

As for the log-ratios, we also computed the weights of logit-ratios and ΔΔ Ct.

All these values were analyzed using a two-step procedure. First, we ran intercept-only linear mixed models (LMM) to test if values of the evolved lines were different from the ancestral population at day 14 post-infection. We considered that if 95% confidence intervals of the intercept did not overlap zero, there was a statistically significant difference between ancestral and evolved lines. These models included the replicated line id as a random factor and the scaled weights:

5 $$w_{scaled} = \frac{{w_{logratio} }}{{w_{mean} }}$$

Second, we ran LMMs to test if log-ratios (or logit-ratios) were different between SI-lines and COI-lines. These models included the replicated line id and, for traits with repeated measurements per individual, the mouse id nested within the replicated line id as random effects, the treatment (SI-lines or COI-lines), the passage (5 or 7), time post Py infection (as a factor), and the two-way and three-way interactions as fixed effects. The models also included the scaled weights. Time p.i. was considered as a factor to allow for non-linear relationships. The full models were then simplified by comparing the AICc values (down to the only-intercept model). The model with the smallest AICc value was considered as the best one. When the Δ AICc value between two competing models was < 2, we proceeded with a model averaging and reported the averaged parameter estimates with the 95% confidence intervals. For the selected minimal models, we computed the marginal and conditional R^2^ [47], which describe the fraction of the variance explained by the fixed effects alone and by the fixed + random effects, respectively.

Differences in survival between mice infected with the ancestral population (data coming from [21]), and mice infected with the evolved lines were analyzed using the Kaplan Meier estimator and significance assessed using a Log-Rank test. Adjustment for multiple comparisons for the Log-Rank test was done using Sidak adjusted *p* values.

For the comparison of parasitemia of mice infected with COI-lines during single infection trials (mismatched environments) or coinfection trials (matched environments), we used a generalized linear mixed model (GLMM) with a beta-distribution of errors (the dependent variable being the proportion of iRBC). The model included the replicated line id and mouse id nested within the replicated line id as random effects and environment (matched or mismatched), time post Py infection (as a factor), and the two-way interaction as fixed effects. As described above, we started with the full model and then selected the best model based on AICc values.

Not all response variables were measured for each mouse at each passage, nor at each time p.i., contributing to differences in sample size across models (for instance, plasma levels of EPO were only measured on a subset of mice at day 14 p.i. after passage 7). In addition, host removal due to the reaching of the end point and technical issues during the processing of the samples also contributed to differences in sample size across response variables.

All the statistical analyses and figures were done using SAS Studio.

## Results

### Disease severity in evolved Py lines

**Fig. 2:**
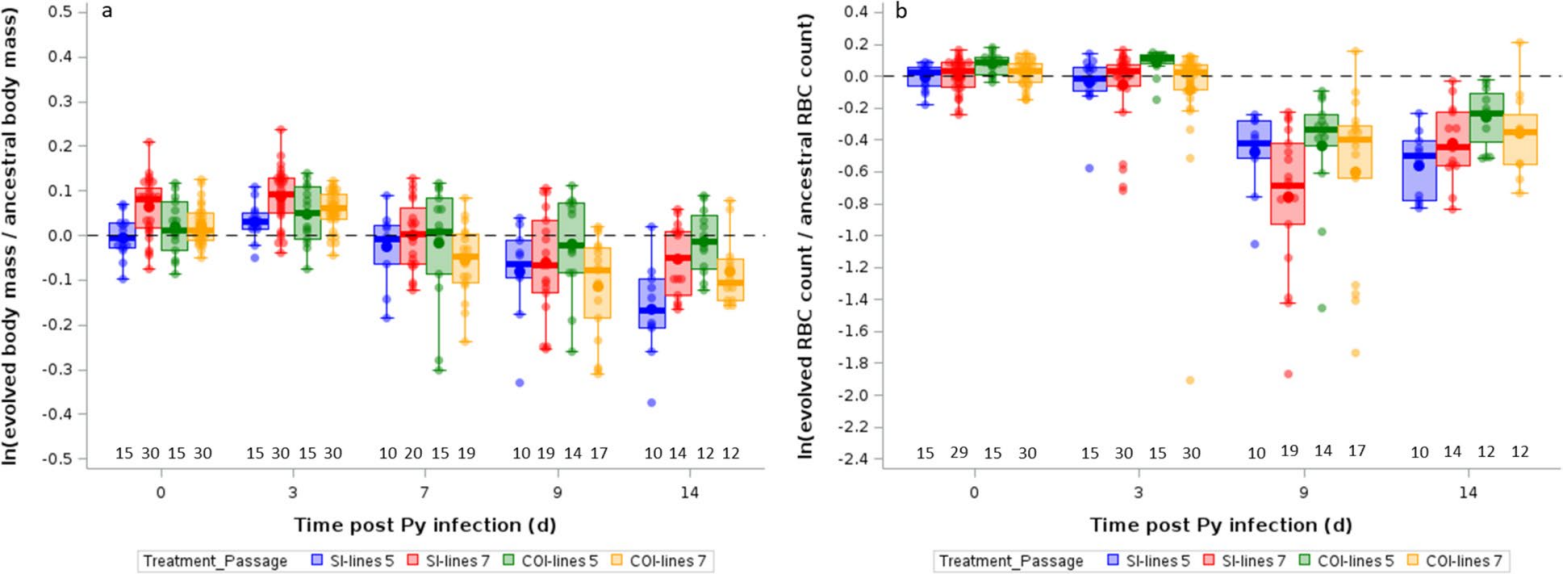
**a** Log-ratios of body mass for mice infected with SI-lines or COI-lines after 5 or 7 passages. **b** Log-ratios of RBC counts for mice infected with SI-lines or COI-lines after 5 or 7 passages. Log-ratios are computed as ln (y_evolved_/mean_ancestral_). The dotted line represents no change with respect to hosts infected with the ancestral Py population. Dots represent the raw data, the boxes represent the interquartile range (IQR), the horizontal lines the median, and whiskers the range of data within 1.5 the IQR. Numbers just above the x-axis represent the sample size per group

Body mass loss is an integrative proxy of disease severity over the course of malaria infection. We first assessed whether mice infected with the evolved lines lost more mass at day 14 p.i. (approximately when parasitemia is at its peak) compared to mice infected with the ancestral population. This was achieved by running an intercept-only LMM. Since the intercept was negative and the 95% confidence intervals did not overlap zero [intercept (95% CI) = − 0.096 (− 0.164/− 0.028), n = 48, Fig. 2a], we considered that mice infected with the evolved lines lost more mass compared to mice infected with the ancestral population. We then ran a LMM with the aim of comparing the changes in body mass of mice infected with SI-lines or COI-lines. The model selection procedure showed that the best model (lowest AICc value, table S3) only included time p.i. (Table 1, Fig. 2a). Therefore, although mice infected with the evolved lines lost more mass compared to mice infected with the ancestral population, there was no difference between the SI-lines and the COI-lines.

**Table 1:** LMM investigating the log-ratios of body mass [ln(*mass*_*evolved*_) – ln(*mean*_*ancestral*_)] up to day 14 p.i. in mice infected with SI-lines or COI-lines after 5 or 7 passages

Fixed effect	Estimate	95% CI	df	F	p
Time p.i			4,258	71.60	< 0.0001
3	0.031	0.020/0.042			
7	− 0.059	− 0.077/− 0.041			
9	− 0.100	− 0.123/− 0.077			
14	− 0.099	− 0.119/− 0.079			

Random effects	Estimate	SE	z	p	
Replicated line id	0.00006	0.0002	0.30	0.3839	
Mouse id * Replicated line id	0.00224	0.0005	4.92	< 0.0001	
Residual	0.00216	0.0002	11.30	< 0.0001	

We started with the full model that included the following fixed effects: treatment [SI-lines (reference) or COI-lines], passage [5 (reference) or 7], time p.i. [0 (reference), 3, 7, 9, 14], and the two- and three-way interactions. The model also included the replicated line id and the mouse id nested within the replicated line id as random effects, and the scaled weights as a weight variable. The model was then simplified based on the AICc values. The model with the lowest AICc value only included time p.i. as fixed effect. No other model was within the range of Δ AICc < 2. We report the parameter estimates with the 95% confidence intervals (CI), degrees of freedom (df), F and *p* values for the fixed effect of the minimal model. For the random effects, we report the parameter estimates with the standard errors (SE), the z and *p* values. N = 7 replicated lines, 90 individuals and 352 observations. Marginal and conditional R^2^ of the minimal model were 34.19% and 68.1%, respectively

The asexual reproduction of *Plasmodium* within the vertebrate host causes anemia due to the lysis of RBC. As for changes in body mass, mice infected with the evolved Py lines lost significantly more RBC compared to mice infected with the ancestral Py population at day 14 p.i. [intercept-only LMM: intercept (95% CI) = − 0.413 (− 0.540/− 0.286), n = 48, Fig. 2b]. The model selection procedure showed that the best model included two fixed effects: the treatment and the time post-infection (table S3, Table 2). Two other models had almost identical AICc values. However, the averaged model showed that only treatment and time p.i. had parameter estimates whose 95% CI did not overlap zero (table S4). The parameter estimates and visual inspection of Fig. 2b showed that log-ratios of RBC counts became negative over the course of the infection and that mice infected with SI-lines had lower log-ratios compared to mice infected with COI-lines (Fig. 2b, Table 2).

**Table 2:** LMM investigating the log-ratios of RBC counts [ln(*rbc*_*evolved*_) – ln(*mean*_*ancestral*_) up to day 14 p.i. in mice infected with SI-lines or COI-lines after 5 or 7 passages

Fixed effects	Estimate	95% CI	df	F	p
Treatment			1,194	11.60	0.0008
COI-lines	0.062	0.026/0.099			
Time p.i			3,194	98.12	< 0.0001
3	− 0.005	− 0.042/0.032			
9	− 0.569	− 0.663/− 0.474			
14	− 0.412	− 0.475/− 0.349			

Random effects	Estimate	SE	z	p	
Replicated line id	0				
Mouse id * Replicated line id	0.0023	0.0012	1.89	0.0296	
Residual	0.0149	0.0015	9.87	< 0.0001	

We started with the full model that included the following fixed effects: treatment [SI-lines (reference) or COI-lines], passage [5 (reference) or 7], time p.i. [0 (reference), 3, 9, 14], and the two- and three-way interactions. The model also included the replicated line id and the mouse id nested within the replicated line id as random effects, and the scaled weights as a weight variable. The model was then simplified based on the AICc values. The model with the lowest AICc value included the treatment and the time p.i. as fixed effects. Two other models were within the range of Δ AICc < 2. The averaged model showed that only treatment and time p.i. had parameter estimates that did not overlap zero. We report the parameter estimates with the 95% confidence intervals (CI), degrees of freedom (df), F and *p* values for the fixed effects of the minimal model. For the random effects, we report the parameter estimates with the standard errors (SE), the z and *p* values. N = 7 replicated lines, 90 individuals and 287 observations. Marginal and conditional R^2^ of the minimal model were 49.93% and 56.65%, respectively

### Infection dynamics in hosts infected with the evolved Py lines

**Fig. 3:**
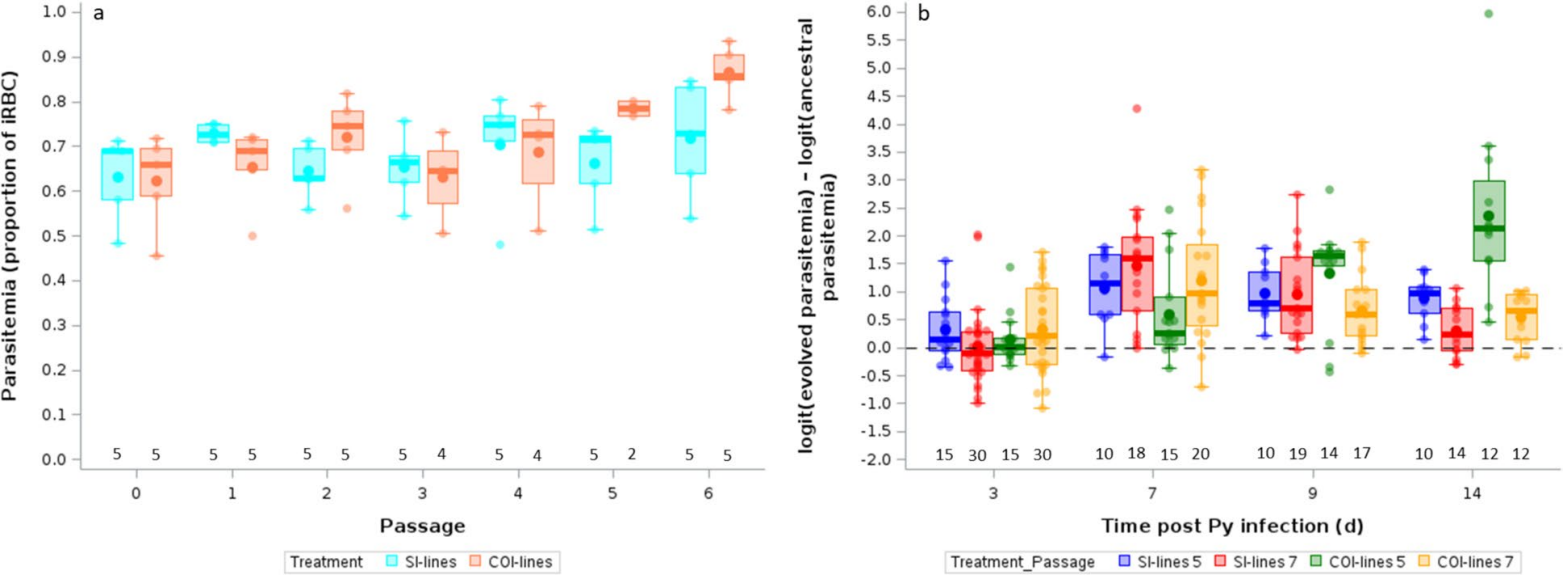
**a** Parasitemia (proportion of iRBC) at day 14 p.i. during the passages (up to passage 6) in single infected (SI-lines) or coinfected (COI-lines) hosts. **b** Logit-ratios of parasitemia for mice infected with SI-lines or COI-lines after 5 or 7 passages. Logit-ratios are computed as logit(y_evolved_) – logit(mean_ancestral_). The dotted line represents no change with respect to hosts infected with the ancestral Py population. For both panels, dots represent the raw data, the boxes represent the interquartile range (IQR), the horizontal lines the median, and whiskers the range of data within 1.5 the IQR. Numbers just above the x-axis represent the sample size per group

Serial passages are supposed to promote the spread of parasite genotypes with the fastest replication rate. To investigate this, we compared the parasitemia at day 14 p.i. over the passages. This was done up to passage 6. Indeed, given that at passage 7 there were only two lines remaining in the coinfection treatment (see below), parasitemia was assessed at day 7 p.i., as to minimize the risk of losing any additional line. We ran a GLM with a beta-distribution of errors and found that parasitemia increased over the passages, and the rate of increase was higher for lines passaged in coinfected hosts compared to lines passaged in single infected hosts, as shown by the interaction between passage and line (the full model being the one with the lowest AICc value, and no model had a Δ AIC < 2; Table 3, Fig. 3a, table S3).

**Table 3:** GLM with a beta-distribution of errors investigating the changes in parasitemia (proportion of iRBC) at day 14 p.i. during the passages (up to passage 6) in single infected or coinfected hosts

Effects	Estimate	95% CI	df	F	p
Treatment			1,61,.61	1.46	0.2315
COI-lines	− 0.215	− 0.572/0.141			
Passage	0.035	− 0.036/0.105	1,61	16.09	0.0002
Treatment x Passage			1,61	7.21	0.0093
COI-lines	0.140	0.036/0.245			

The full model included the treatment [SI-lines (reference) or COI-lines], passage (from 0 to 6, as a continuous variable), and the two-way interaction as explanatory variables. The model selection procedure showed that the full model had the smallest AICc value and all other models had Δ AICc values > 2. We report the parameter estimates with the 95% confidence intervals (CI), degrees of freedom (df), F and *p* values. N = 65 observations. Pseudo-R^2^ was computed according to Cox and Snell [12] and was equal to 28%

This result does not necessarily show that parasitemia increased faster for Py lines evolving in coinfected hosts because here the two groups differed both in terms of the selection history and the current environment (single infection or coinfection). We therefore used these evolved lines to infect mice during single infection evaluation trials, as to isolate the effect of the selection history. The intercept-only model ran on day 14 p.i. showed that mice infected with the evolved lines had higher parasitemia compared to mice infected with the ancestral population [intercept-only LMM: intercept (95% CI) = 0.673 (0.375/0.972), n = 48, Fig. 3b]. We then ran the model comparing SI-lines and COI-lines. The model selection procedure showed that the full model had the lowest AICc value (table S3) and the three-way interaction between treatment x passage x time p.i. was statistically significant (Table 4, Fig. 3b). Visual inspection of Fig. 3b suggests that this pattern was due to COI-lines evaluated after passage 5, for which the difference in parasitemia with respect to the ancestral population linearly increased over the course of the infection, while for the other groups the logit-ratios were similarly above 0 from day 7 p.i. onwards.

**Table 4:** LMM investigating the logit-ratios of parasitemia [logit(*parasitemia*_*evolved*_) – logit(*mean*_*ancestral*_)] up to day 14 p.i. in mice infected with SI-lines or COI-lines after 5 or 7 passages

Fixed effects			Estimate	95% CI	df	F	p
Treatment					1,159	1.30	0.2551
COI-lines			− 0.156	− 0.602/0.290			
Passage	7		− 0.292	− 0.669/0.085	1,159	5.50	0.0203
Time p.i					3,159	38.83	< 0.0001
		7	0.704	0.287/1.120			
		9	0.612	0.243/0.982			
		14	0.516	0.096/0.937			
Treatment x Passage					1,159	3.01	0.0846
COI-lines	7		0.433	− 0.086/0.951			
Treatment x Time p.i					3,159	6.86	0.0002
COI-lines		7	− 0.242	− 0.813/0.329			
COI-lines		9	0.638	0.059/1.217			
COI-lines		14	1.833	0.980/2.686			
Passage x Time p.i					3,159	11.93	< 0.0001
	7	7	0.775	0.157/1.392			
	7	9	0.313	− 0.203/0.829			
	7	14	− 0.211	− 0.774/0.352			
Treatment x Passage x Time p.i					3,159	5.98	0.0007
COI-lines	7	7	− 0.364	− 1.222/0.494			
COI-lines	7	9	− 1.183	− 1.947/− 0.419			
COI-lines	7	14	− 1.840	− 2.857/− 0.822			

Random effects			Estimate	SE	z	p	
Replicated line id			0.021	0.023	0.91	0.1815	
Mouse id * Replicated line id			0.100	0.036	2.76	0.0029	
Residual			0.332	0.036	9.15	< 0.0001	

We started with the full model that included the following fixed effects: treatment [SI-lines (reference) or COI-lines], passage [5 (reference) or 7], time p.i. [3 (reference), 7, 9, 14], and the two- and three-way interactions. The model also included the replicated line id and the mouse id nested within the replicated line id as random effects, and the scaled weights as a weight variable. The model was then simplified based on the AICc values. The model with the lowest AICc value was the full model. No other model was within the range of Δ AICc < 2. We report the parameter estimates with the 95% confidence intervals (CI), degrees of freedom (df), F and *p* values for the fixed effects. For the random effects, we report the parameter estimates with the standard errors (SE), the z and *p* values. N = 7 replicated lines, 90 individuals and 261 observations. Marginal and conditional R^2^ of the model were 31.14% and 49.54%, respectively

### Tolerance to the infection in hosts infected with the evolved Py lines

**Fig. 4:**
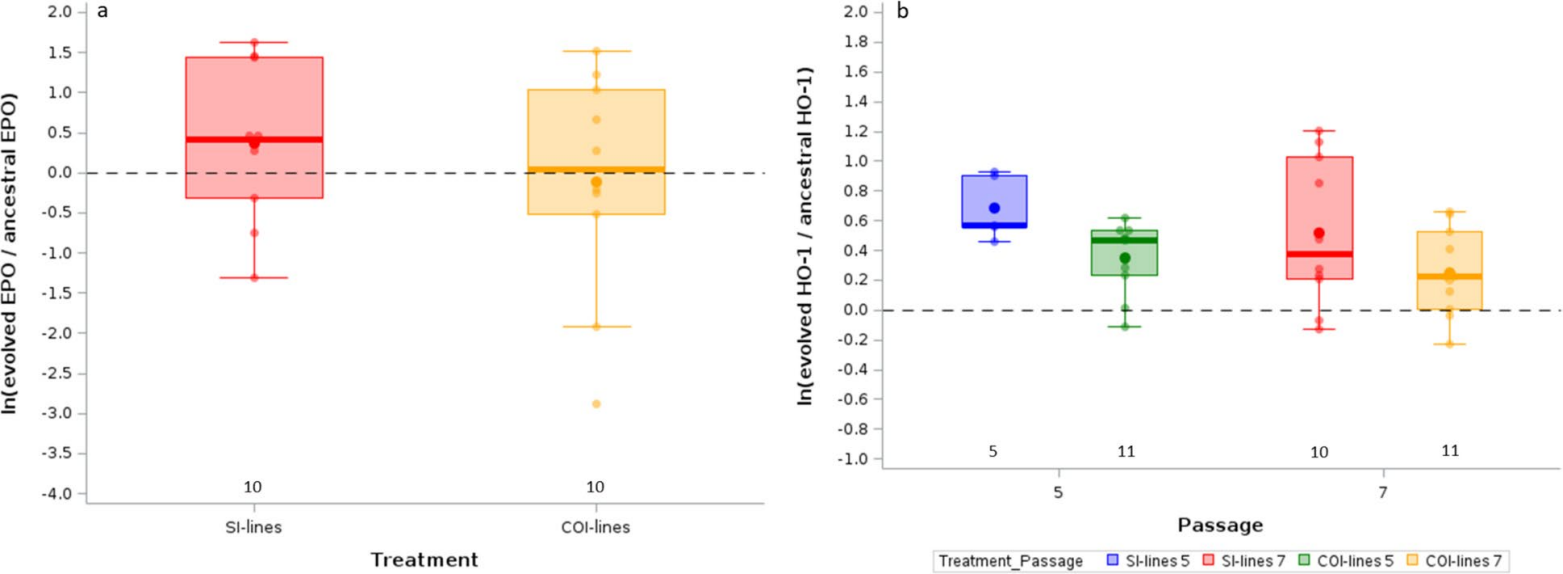
**a** Log-ratios of plasma EPO levels at day 14 p.i. for mice infected with SI-lines or COI-lines after 7 passages. **b** Log-ratios of plasma HO-1 levels at day 14 p.i. for mice infected with SI-lines or COI-lines after 5 or 7 passages. Log-ratios are computed as ln(y_evolved_/mean_ancestral_). The dotted line represents no change with respect to hosts infected with the ancestral Py population. Dots represent the raw data, the boxes represent the interquartile range (IQR), the horizontal lines the median, and whiskers the range of data within 1.5 the IQR. Numbers just above the x-axis represent the sample size per group

Severity of disease symptoms does not depend only on the damage caused by parasite replication, but also on the capacity of the host to repair it [45]. As mentioned above, *Plasmodium* replication causes the lysis of RBC and induces anemia. Lost RBC can, however, be replaced by hematopoiesis, reducing the net cost of the infection. We therefore assessed whether mice infected with the evolved lines after 7 passages differed in the amount of circulating erythropoietin, the hormone regulating the production of RBC, at day 14 post Py infection. An intercept-only LMM showed that there was no difference between the ancestral and the evolved lines [intercept (95% CI) = 0.219 (− 0.646/1.085)]. Similarly, there was no difference between the SI-lines and the COI-lines (LMM, F_1,15_ = 0.60, p = 0.4514, Fig. 4a; and the Δ AIC between the intercept-only model and the model including the treatment was < 2, table S3).

Lysis of RBC releases free heme groups that have strong pro-oxidant activities and have been shown to be an important component of *Plasmodium* pathogenesis [39, 54]. We therefore investigated whether evolved Py lines elicited a different response in terms of the production of the scavenging enzyme heme oxygenase-1 (HO-1) at day 14 post-infection. We found that, overall, hosts infected with the evolved lines had a much stronger response in terms of HO-1, compared to mice infected with the ancestral Py population [intercept-only LMM, intercept (95% CI) = 0.492 (0.270/0.714), n = 37]. The LMM comparing the evolved lines showed that COI-lines induced a weaker HO-1 response compared to SI-lines (LMM, F_1,30_ = 9.30, p = 0.0048; Fig. 4b). This model had the lowest AICc value (table S3). However, the additive model that included treatment and passage as fixed effects had a Δ AICc < 2 (table S3). The averaged model showed that only the treatment had a parameter estimate with 95% CI non overlapping zero (table S5).

**Fig. 5:**
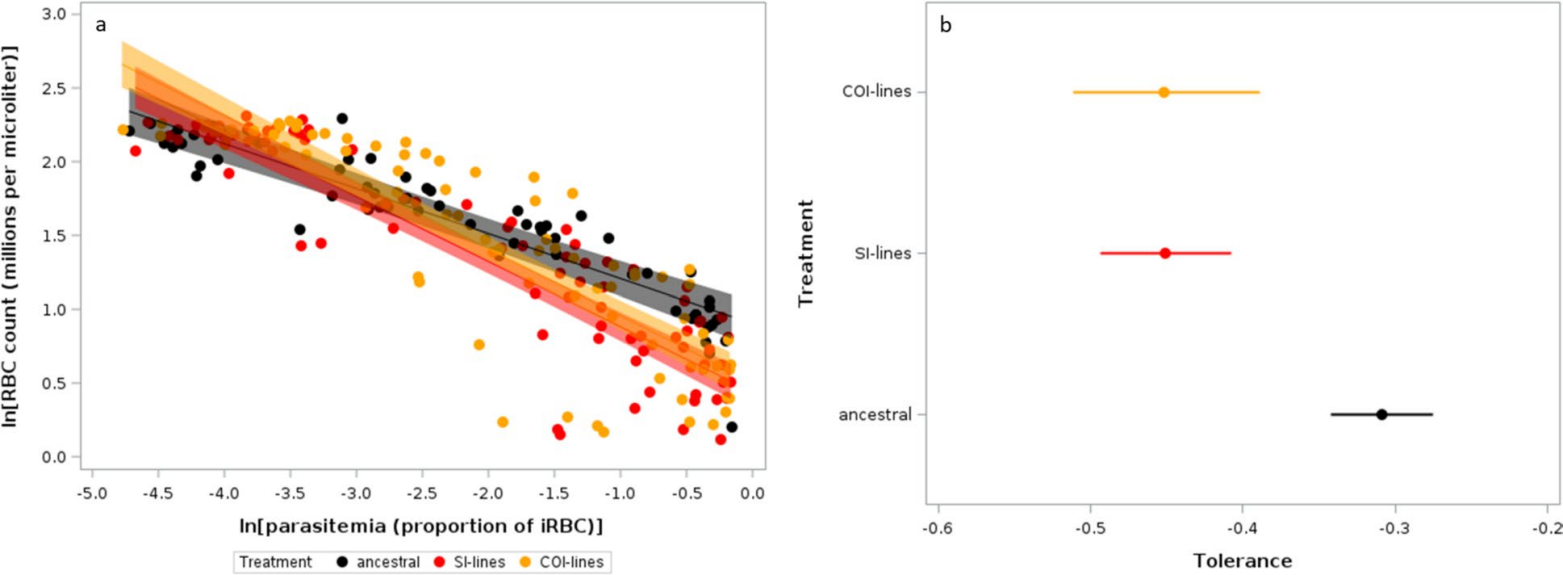
**a** Log–log plot of the relationship between RBC counts and parasitemia in hosts infected with the ancestral Py population or with the evolved lines (SI-lines, COI-lines) after 7 passages. The dots represent the raw data, the line and the shaded area the LMM fit with the 95% CI. **b** Tolerance to the Py infection in mice infected with the ancestral population or the evolved (SI-lines, COI-lines) lines. Tolerance is expressed as the slope (with the 95% CI) of the log–log regression of RBC counts on parasitemia

Whatever the possible mechanisms underlying the host capacity to minimize the cost of the infection, a more direct test of the difference in tolerance between groups is to regress a proxy of the cost of infection on parasitemia [50],the steeper the slope of the regression, the weaker the tolerance. We found that mice infected with the evolved lines after 7 passages were less tolerant to Py infection (steeper slope between RBC counts and parasitemia) compared to mice infected with the ancestral population (Table 5; Fig. 5a). However, tolerance did not differ between mice infected with SI-lines or COI-lines (Fig. 5b). The full model with the interaction between treatment and parasitemia had the lowest AICc value, and no model had a Δ AICc < 2 (Table S3).

**Table 5:** LMM investigating the relationship between ln(RBC counts) and ln(parasitemia) in mice infected with Py from the ancestral population, with SI-lines or COI-lines

Fixed effects	Estimate	95% CI	df	F	p
Treatment			2,142	11.39	< 0.0001
SI-lines	− 0.462	− 0.662/− 0.262			
COI-lines	− 0.396	− 0.601/− 0.190			
Parasitemia	− 0.305	− 0.357/− 0.253	1,142	765.78	< 0.0001
Treatment x Parasitemia			2,142	10.16	< 0.0001
SI-lines	− 0.136	− 0.205/− 0.067			
COI-lines	− 0.146	− 0.219/− 0.074			

Random effect	Estimate	SE	z	p	
Mouse id	0.017	0.009	1.90	0.0287	
Residual	0.084	0.010	8.47	< 0.0001	

We started with the full model that included the following fixed effects: treatment [ancestral (reference), SI-lines, COI-lines], parasitemia (continuous variable), and the two-way interaction. The model also included the mouse id as a random effect. The model was then simplified based on the AICc values. The model with the lowest AICc value was the full model. No other model was within the range of Δ AICc < 2. We report the parameter estimates with the 95% confidence intervals (CI), degrees of freedom (df), F and *p* values for the fixed effects. For the random effect, we report the parameter estimates with the standard errors (SE), the z and *p* values. N = 75 individuals and 220 observations. Marginal and conditional R^2^ of the model were 73.34% and 81.09%, respectively

### Virulence of the evolved Py lines

**Fig. 6:**
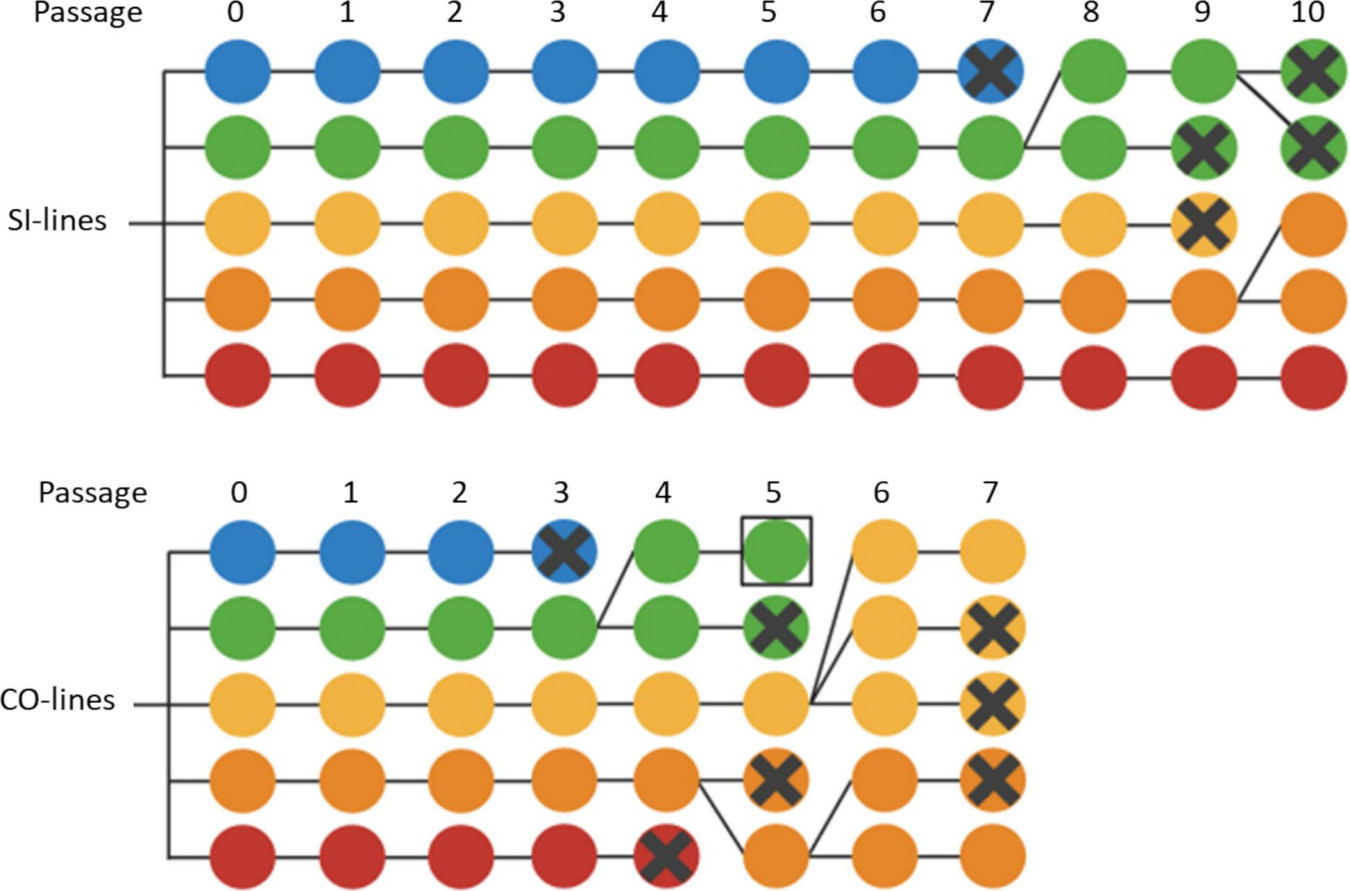
Transmission of Py across passages for the two treatments (SI-lines and COI-lines). Each circle corresponds to a host and each color to a Py line. Crosses indicate pre-transmission host mortality and therefore the loss of the line. When one line was lost, the host meant to be infected with the lost line was infected with one of the remaining lines. For instance, the blue line of the COI treatment was lost at passage 3, and the host meant to be infected with the blue line was instead infected with the green line. Up to passage 7, one line was lost among the SI-lines (the blue line) and three among the COI-lines (blue, green and red). Up to passage 10, three lines were lost among the SI-lines (the COI treatment having been stopped at passage 7). The square around the green circle at passage 5 for the COI treatment indicates a host where Py did not replicate, possibly due to a failure during the Py injection

**Fig. 7:**
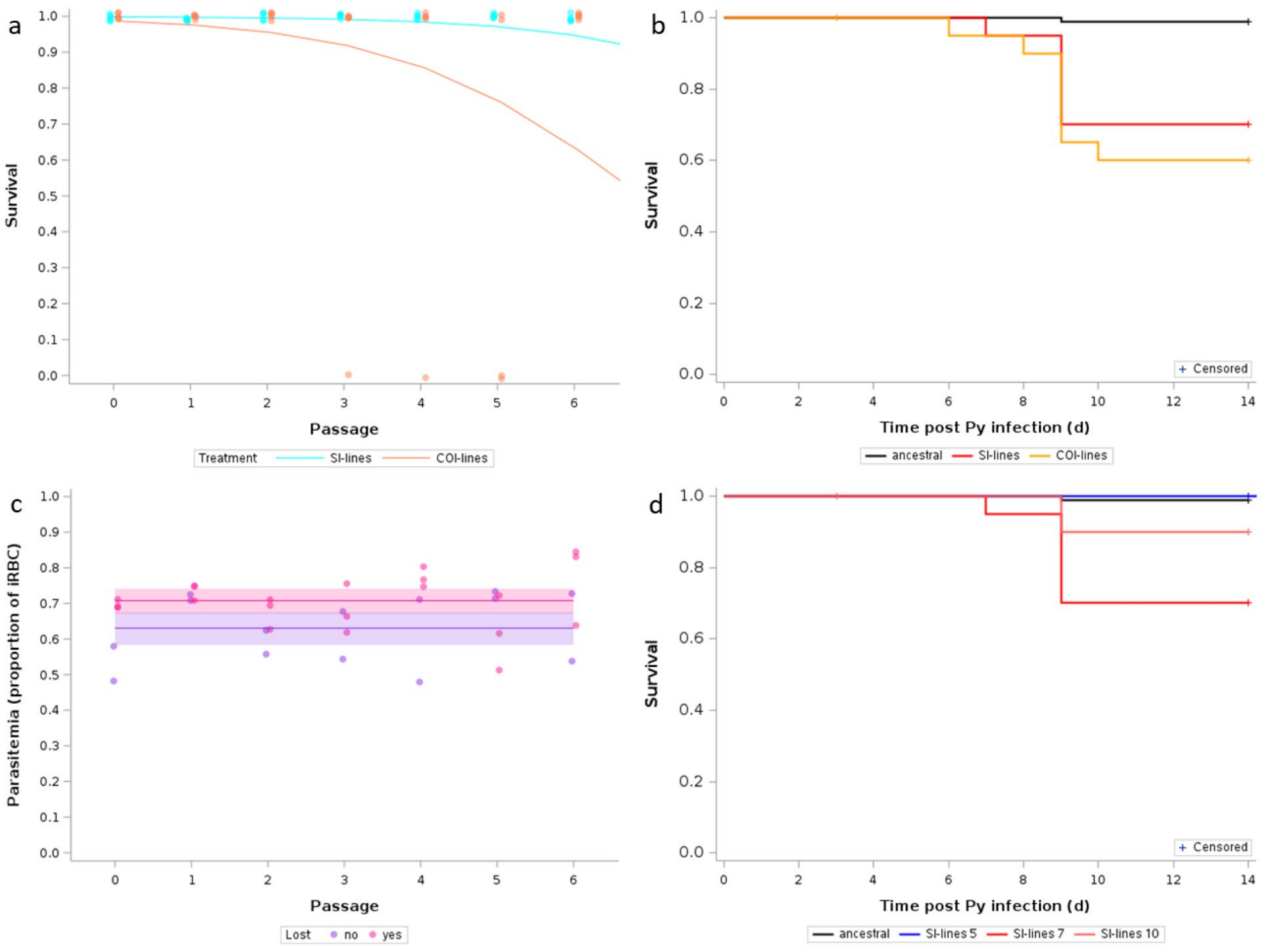
**a** Proportion of single infected and coinfected hosts alive at day 14 p.i. over the passages. The dots represent the raw data (1 = alive, 0 = dead) and the curves represent the fit of a GLM with a binomial distribution or errors. N = 5 hosts per treatment and passage, except for passage 5 and treatment COI for which n = 4. **b** Survival up to day 14 p.i. of hosts infected with the ancestral Py population (n = 94, data from [21]), with the SI-lines (n = 30) or the COI-lines (n = 30) after 7 passages. Crosses indicate censored observations. **c** Parasitemia (proportion of iRBC) at day 14 p.i. of hosts in the single infection treatment, according to the fate of the lines after 10 passages [pink = lines that were lost (n = 3); violet = lines that were not lost (n = 2)]. Dots represent the raw data, the lines and the shaded area the GLM fit with the 95% CI. **d** Survival up to day 14 p.i. of hosts infected with the ancestral Py population (n = 94, data from [21]) or with SI-lines after 5 passages (n = 15), 7 passages (n = 30), 10 passages (n = 15). Crosses indicate censored observations

During the passages, Py was transmitted from mouse to mouse at day 14 p.i., and due to host mortality occurring before transmission, some of the Py lines were lost. Up to passage 7, 1 line (out of five) was lost in the single infection treatment, whereas 3 lines (out of five) were lost in the coinfection treatment (Fig. 6) (we did not take into account here the line lost during the revitalization phase, because this was due to the failure of Py to replicate). Pre-transmission host mortality increased over the passages (GLM with a binomial distribution of errors: χ^2^ = 13.15, p = 0.0003, n = 39) and was higher for the COI-lines (χ^2^ = 9.08, p = 0.0026, n = 39) (one mouse was excluded because the parasitemia at day 14 p.i. was nil suggesting that the transmission had failed) (Fig. 7a). When tested in single infection evaluation trials, we found that both evolved lines elicited higher host mortality than the ancestral population (Log-Rank test, χ^2^_2_ = 31.22, p < 0.0001, Fig. 7b; both pairwise comparisons, Sidak adjusted p’s < 0.0001); however, there was no difference between the SI-lines and the COI-lines (Sidak adjusted p = 0.6688, Fig. 7b).

For the single infection passages, Py was transmitted up to passage 10. This allowed us to check whether the cost of virulence (pre-transmission host mortality) also occurred in this group over a higher number of passages. Indeed, the number of Py lines lost kept increasing up to passage 10 (60% of lines were lost, Fig. 6), as did mortality (GLM with a binomial distribution of errors: χ^2^ = 29.91, p < 0.0001, n = 55, figure S2). In agreement with the hypothesis that the lines with the fastest replication were the most virulent and more likely to be lost during the passages, we found that up to passage 6 (before any line had been lost) the parasitemia at day 14 p.i. was higher for lines that were subsequently lost compared to lines that were not lost (F_1,33_ = 7.49, p = 0.0099, Fig. 7c). We also found that after ten passages, the persisting lines that were used for the evaluation trials had lower virulence compared to the lines tested after seven passages (Sidak adjusted p = 0.0055; Fig. 7d), but still higher than the ancestral population (Sidak adjusted p = 0.0363; Fig. 7d). Therefore, by transmitting Py at day 14 p.i., we allowed selection to operate against the most virulent lines.

### Immune response of hosts infected with the evolved Py lines

**Fig. 8:**
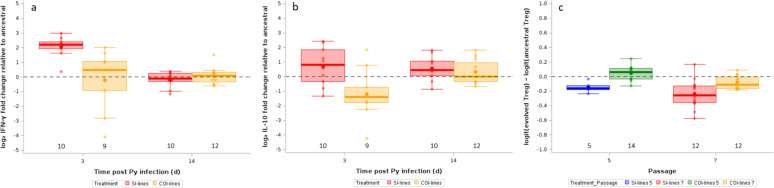
**a** Log_2_ fold change of *IFN-γ* mRNA in spleen at day 3 and 14 p.i. in hosts infected with SI-lines or COI-lines after 7 passages relative to expression in mice infected with the ancestral Py population. **b** log_2_ fold change of *IL-10* mRNA in spleen at day 3 and 14 p.i. in hosts infected with SI-lines or COI-lines after 7 passages relative to expression in mice infected with the ancestral Py population. **c** Logit-ratios of the proportion of FoxP3^+^ cells within CD4^+^ T cells at day 14 p.i. for mice infected with SI-lines or COI-lines after 5 or 7 passages. Logit-ratios are computed as logit(y_evolved_) – logit(mean_ancestral_). The dotted line represents no change with respect to hosts infected with the ancestral Py population. Dots represent the raw data, the boxes represent the interquartile range (IQR), the horizontal lines the median, and whiskers the range of data within 1.5 the IQR. Numbers just above the x-axis represent the sample size per group

Infection with *Plasmodium* induces a strong pro-inflammatory response which, in turn, induces the production of regulatory mechanisms as to resolve inflammation. We therefore wished to test whether evolved Py lines elicited different pro- and anti-inflammatory responses in mice compared to the ancestral Py population. To this aim, we assessed the gene expression of the pro-inflammatory cytokine IFN-γ and the anti-inflammatory cytokine IL-10 in splenocytes at day 3 and 14 post Py infection. Overall, the gene expression (ΔΔ Cts) of the evolved lines did not differ from the ancestral population, as shown by an intercept-only LMM [IFN-γ, intercept (95% CI) = − 0.074 (− 0.429/0.281); IL-10, intercept (95% CI) = − 0.348 (− 0.869/0.173)]. However, after 7 passages, SI-lines elicited an upregulated expression of IFN-γ compared to COI-lines at day 3 p.i., but this difference vanished by day 14 p.i. (Table 6, Fig. 8a). This result was mirrored by the findings on the gene expression of the regulatory cytokine IL-10. SI-lines tended to elicit an upregulated expression of IL-10 compared to COI-lines at day 3 p.i., and at day 14 p.i. the two groups had identical ΔΔ Ct values (Table 7, Fig. 8b). For both IFN-γ and IL-10 gene expression the full model had the lowest AICc value, with no other model having a Δ AICc < 2 (table S3).

**Table 6:** LMM investigating IFN-γ gene expression (ΔΔ Ct relative to ancestral) at day 3 and 14 p.i. in mice infected with SI-lines or COI-lines after 7 passages

Fixed effects		Estimate	95% CI	df	F	p
Treatment				1,34	6.71	0.0140
COI-lines		2.276	0.782/3.770			
Time p.i	14	2.259	1.355/3.164	1,34	6.78	0.0136
Treatment x Time p.i				1,34	11.11	0.0021
COI-lines	14	− 2.537	− 4.083/− 0.990			

Random effect		Estimate	SE	z	p	
Replicated line id		0.008	0.056	0.13	0.4465	
Residual		0.423	0.103	4.11	< 0.0001	

We started with the full model that included the following fixed effects: treatment [SI-lines (reference) or COI-lines], time p.i. [3 (reference), 14], and the two-way interaction. The model also included the replicated line id as a random effect, and the scaled weights as a weight variable. Mouse identity was not included in the model, because each mouse provided a single ΔΔ Ct measurement. The model was then simplified based on the AICc values. The model with the lowest AICc value was the full model. No other model was within the range of Δ AICc < 2. We report the parameter estimates with the 95% confidence intervals (CI), degrees of freedom (df), F and *p* values for the fixed effects. For the random effect, we report the parameter estimates with the standard errors (SE), the z and *p* values. N = 5 replicated lines and 41 observations. Marginal and conditional R^2^ of the model were 38.98% and 40.05%, respectively

**Table 7:** LMM investigating IL-10 gene expression (ΔΔ Ct relative to ancestral) at day 3 and 14 p.i. in mice infected with SI-lines or COI-lines after 7 passages

Fixed effects		Estimate	95% CI	df	F	p
Treatment				1,34	6.87	0.0130
COI-lines		1.902	0.393/3.411			
Time p.i	14	0.151	− 0.932/1.233	1,34	2.74	0.1069
Treatment x Time p.i				1,34	4.10	0.0507
COI-lines	14	− 1.651	− 3.301/0.005			

Random effect		Estimate	SE	z	p	
Replicated line id		0.003	0.117	0.03	0.4895	
Residual		0.956	0.233	4.10	< 0.0001	

We started with the full model that included the following fixed effects: treatment [SI-lines (reference) or COI-lines], time p.i. [3 (reference), 14], and the two-way interaction. The model also included the replicated line id as a random effect, and the scaled weights as a weight variable. Mouse identity was not included in the model, because each mouse provided a single ΔΔ Ct measurement. The model was then simplified based on the AICc values. The model with the lowest AICc value was the full model. No other model was within the range of Δ AICc < 2. We report the parameter estimates with the 95% confidence intervals (CI), degrees of freedom (df), F and *p* values for the fixed effects. For the random effect, we report the parameter estimates with the standard errors (SE), the z and *p* values. N = 5 replicated lines and 41 observations. Marginal and conditional R^2^ of the model were 17.53% and 17.79%, respectively

Finally, we assessed whether the evolved lines elicited a different expansion of regulatory T cells in the spleen at day 14 p.i. and found that, overall, hosts infected with the evolved lines had a lower proportion of FoxP3^+^ cells within CD4^+^ lymphocytes [intercept-only LMM, intercept (95% CI) = − 0.115 (− 0.209/− 0.020), n = 43]. The comparison between evolved lines indicated that COI-lines elicited a stronger expansion of the population of Treg cells compared to the SI-lines (Table 8, Fig. 8c). The additive model with treatment + passage had the lowest AICc value, and no model had a Δ AICc < 2 (table S3).

**Table 8:** LMM investigating the logit-ratios of the proportion of FoxP3^+^ cells within CD4^+^ lymphocytes [logit(Treg_*evolved*_) – logit(*mean*_*ancestral*_)] at day 14 p.i. in mice infected with SI-lines or COI-lines after 5 or 7 passages

Fixed effects		Estimate	95% CI	df	F	p
Treatment				1,35	19.99	< 0.0001
COI-lines		0.190	0.104/0.276			
Passage	7	− 0.125	− 0.191/− 0.058	1,35	14.57	0.0005

Random effect		Estimate	SE	z	p	
Replicated line id		0.0008	0.0018	0.44	0.3286	
Residual		0.0108	0.0025	4.29	< 0.0001	

We started with the full model that included the following fixed effects: treatment [SI-lines (reference) or COI-lines], passage [5 (reference) or 7], and the two-way interaction. The model also included the replicated line id as a random effect, and the scaled weights as a weight variable. Mouse identity was not included in the model, because each mouse provided a single Treg measurement. The model was then simplified based on the AICc values. The additive model (treatment + passage) had the lowest AICc value. No other model was within the range of Δ AICc < 2. We report the parameter estimates with the 95% confidence intervals (CI), degrees of freedom (df), F and *p* values for the fixed effects. For the random effect, we report the parameter estimates with the standard errors (SE), the z and *p* values. N = 7 replicated lines and 43 observations. Marginal and conditional R^2^ of the model were 44.19% and 48.06%, respectively

### Effect of matched vs mismatched environments on Plasmodium adaptation

We also evaluated COI-lines in coinfected hosts, which allowed us to investigate whether Py performance changed according to the mismatch between the environment encountered during the passages (coinfected hosts) and the environment faced during the evaluation trials (either single infected or coinfected hosts).

**Fig. 9:**
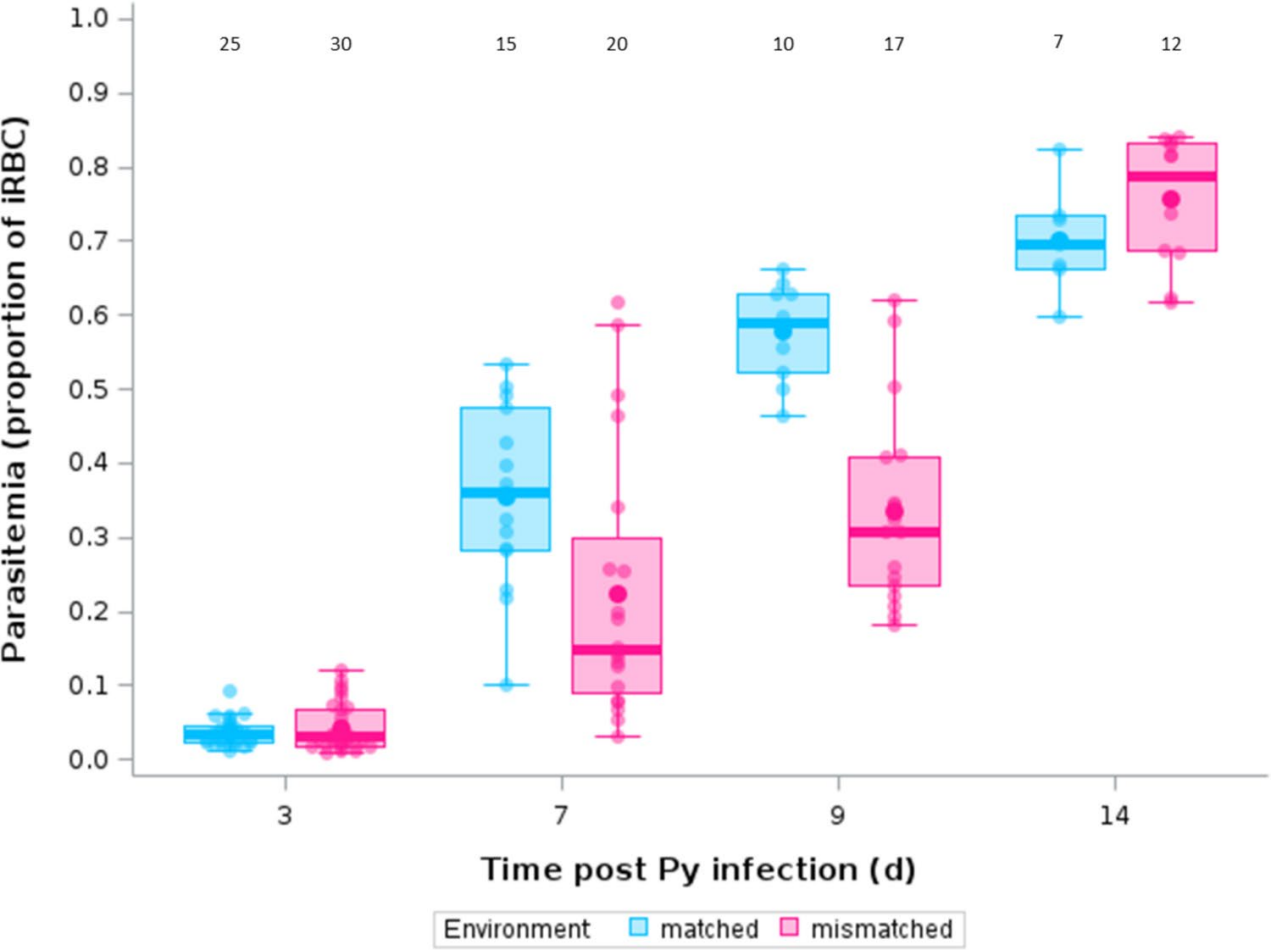
Parasitemia (proportion of iRBC) over the course of the infection in hosts infected with COI-lines after 7 passages that experienced matched environments between the passages and the evaluation trials (coinfection/coinfection) or mismatched environments (coinfection/single infection). Dots represent the raw data, the boxes represent the interquartile range (IQR), the horizontal lines the median, and whiskers the range of data within 1.5 the IQR. Numbers at the top of the figure represent the sample size per group

We used parasite replication as a proxy of parasite performance and found that the infection dynamics differed between matched and mismatched environments, as shown by the environment x time p.i. interaction (Table 9). The full model had the lowest AICc value and no model had a Δ AICc < 2 (table S3). In agreement with the prediction, the mismatched environment group had lower parasitemia compared to the matched environment group at day 7 and 9 p.i., with parasitemia converging towards high values at day 14 p.i. for both groups (Fig. 9). However, there was no difference in infection-induced host mortality between matched- and mismatched-environment groups (Log-rank Test, γ^2^ = 0.438, p = 0.5079; figure S3a), and hosts had similar tolerance when infected with matched- or mismatched-environment Py (LMM, environment x parasitemia, F_1,113_ = 1.06, p = 0.3058, n = 55 individuals and 135 observations; figure S3b).

**Table 9:** GLMM with a beta-distribution of errors investigating the changes in parasitemia up to day 14 p.i. in mice infected with COI-lines after 7 passages having encountered matched or mismatched environments between the passages and the evaluation trials

Fixed effects		Estimate	95% CI	df	F	p
Environment				1,75	10.25	0.0020
Mismatched		0.102	− 0.442/0.645			
Time p.i				3,75	228.96	< 0.0001
	7	2.713	2.256/3.171			
	9	3.821	3.332/4.311			
	14	4.473	3.936/5.010			
Environment x Time p.i				3,75	11.25	< 0.0001
Mismatched	7	− 0.844	− 1.450/− 0.238			
Mismatched	9	− 1.287	− 1.916/− 0.659			
Mismatched	14	0.015	− 0.672/0.702			

Random effects		Estimate	SE	z	p	
Replicated line id		0.079	0.088	0.90	0.1839	
Mouse id * Replicated line id		0.074	0.040	1.86	0.0314	

We started with the full model that included the following fixed effects: environment [matched (reference) or mismatched], time p.i. [3 (reference), 7, 9, 14], and the two-way interaction. The model also included the replicated line id and the mouse id nested within the replicated line id as random effects. The model was then simplified based on the AICc values. The full model had the lowest AICc value. No other model was within the range of Δ AICc < 2. We report the parameter estimates with the 95% confidence intervals (CI), degrees of freedom (df), F and *p* values for the fixed effects. For the random effects, we report the parameter estimates with the standard errors (SE), the z and *p* values. The value of the residual variance was fixed to π^2^/3. N = 55 individuals, 2 lines and 136 observations. Marginal and conditional R^2^ of the model were 36.48% and 39.30%, respectively

## Discussion

We showed that serial passages of Py rapidly resulted in faster replication rate and increased virulence; however, SI-lines and COI-lines had similar virulence (and more generally induced disease symptoms of relatively similar severity), and hosts similarly tolerated the infections with the two selection lines when evaluated during single infection trials. At first glance, this might be seen as evidence that environmental (within-host) conditions encountered in single infected and coinfected hosts exerted similar selection pressures on Py, but it is also possible that the cost of virulence incurred by the strains with the fastest replication rate during the passages, constrained virulence evolution. We also found evidence suggesting that Py rapidly adapted to the conditions provided by the host since it had a faster replication rate when the evaluation trials of the COI-lines matched the environment experienced during the passages.

Parasitic microorganisms have the capacity to rapidly adapt to novel environments due to their large population size, short generation time and high mutation rate [20]. Examples of parasite adaptation to novel environments (or novel host genotypes/phenotypes) are manifold and come from a variety of approaches based both on lab experiments and field observations [5, 6, 38, 43, 51, 52, 61].

In our experimental approach, Py did not face a novel host *sensu stricto* but experienced different environmental conditions according to the coinfection status of the host. Indeed, in a previous work, we showed that when Py infects hosts previously infected with Hp, the infection dynamics is altered and virulence of Py increases compared to single infection [21]. The mechanisms underlying the effects of coinfection are essentially related to the regulation of the anti-malarial immune response (top-down regulation), providing a favorable ground for Py replication in coinfected hosts [21]. Given that faster replication rate is correlated to the production of transmissible stages, this result suggests higher Py fitness in coinfected hosts, unless host death occurs prior to transmission.

Therefore, the follow-up question was, does the immune environment provided by a single infected or a coinfected host select for Py with specific replication rate and virulence? To predict the direction of a possible microevolutionary response, we tentatively used the theoretical framework developed by Fenton [24] who predicted that under some specific conditions, deworming should select for increased pathogen virulence. Although the structure of the model differs with many respects from our experimental approach, there are also several analogies between them. In particular, Fenton [24] considered different types of within-host interactions between helminths and microparasites, both antagonistic and synergistic effects. Among these, he explicitly considered two cases that match the interaction between Py and Hp: helminths aggravate the severity of symptoms caused by microparasites, and hosts infected with helminths have a reduced recovery rate. Under these specific conditions, the model predicted that in the absence of helminth infection (e.g., during deworming), microparasite virulence should increase.

Our results did not provide a strong support to this prediction (only RBC loss was slightly higher when mice were infected with SI-lines compared to COI-lines) and, if any, tended rather to indicate that Py passaged in coinfected hosts might have acquired higher virulence compared to lines passaged in single infected mice. This is based on the findings that the lines with the fastest replication rate, inducing a pre-transmission host mortality, were lost during the passages, which occurred more in the coinfection treatment. Therefore, the subset of lines still available for the evaluation trials was probably skewed towards the less virulent ones, especially in the coinfection treatment. It should be reminded that we adopted a transmission rule where Py was passaged at a set (and relatively late) time point, irrespective of the time when symptoms developed. This differs from the other strategy usually implemented in serial passage experiments where pathogens are transmitted before the onset of symptoms (at an early time point) [51]. Passaging pathogens early or late during the infectious period, obviously changes the selection pressures acting on replication rate and virulence. When passages occur early during the infectious period, pathogen strains with the fastest replication rate outnumber slow replicating strains in the inoculum that is transferred between hosts. In addition to this, given that transmission occurs before host death, fast replicating strains do not pay the cost they may pay under natural conditions when hosts may die before transmission.

Therefore, although serial passages are powerful tools to promote parasite adaptation, they poorly replicate the transmission conditions encountered in the wild. Our experimental design while being still far away from the natural conditions, at least allowed the cost of virulence to be expressed. Indeed, we found that parasitemia at the day of transmission increased over the passages and the lines that were finally lost were those that induced the highest parasitemia in the host. In agreement with this idea, we also found that, in the single infection treatment, once the most virulent lines were wiped out after ten passages, Py virulence decreased. Overall, these results nicely illustrate how host mortality prior to transmission can compromise any competitive advantage of fast replicating strains, which is one of the main assumptions of the trade-off model of parasite virulence [14]. Extrapolating results obtained under artificial lab conditions to the real world is never easy, however, with all the necessary caution, our results suggest that although conditions encountered in helminth coinfected hosts might favor the most virulent *Plasmodium* strains, reduced inter-host transmission due to mortality might rapidly offset the selective benefit of the virulent strains.

There are other alternative explanations for the lack of differences between SI-lines and COI-lines during the evaluation trials. First, it is possible that we did not passage Py long enough to observe a divergence between the lines. We stopped the passages for the COI-lines after 7 passages because of the severity of the symptoms which resulted in the loss of 60% of the lines. That said, Py rapidly responded to the passages since the evolved lines (independently of the selection regime) had faster replication rate and induced more severe symptoms. Therefore, it is unlikely that the lack of divergence between the SI-lines and the COI-lines is due to the reduced number of passages. Another possible explanation is merely that Py encountered similar conditions in single infected and coinfected hosts, reducing the scope for selection to differentially operate on replication rate or virulence. However, previous work conducted on this (and similar) system(s) has consistently found that coinfected hosts have different immune profiles compared to single infections, both in terms of specific anti-malarial effectors and immunoregulatory pathways [13, 21, 29, 37, 48, 57, 59]. Therefore, it seems unlikely that Py experienced similar immune selection in single infected and coinfected hosts. That said, we cannot exclude that the strength of selection was not enough to produce a microevolutionary divergence between treatments after 7 passages.

Consistent with the results on the infection dynamics and virulence, we found that the infection with the evolved lines was equally tolerated by the host. We screened a couple of possible mechanisms accounting for variability in tolerance to malaria infection, the capacity to restore lost RBC and the capacity to detoxify free heme [9, 18, 33, 54], and found that hosts infected with the COI-lines had a weaker HO-1 production compared to hosts infected with SI-lines. Although this result might suggest a weaker tolerance of hosts infected with COI-lines, the slope of the regression of RBC counts on parasitemia was similar for hosts infected with SI-lines and COI-lines, showing that for a given intensity of the infection, the cost in terms of RBC loss was the same whatever the treatment. Interestingly, however, we found that evolved Py lines were less well tolerated by the host compared to the ancestral population. This result shows that the passages promoted Py genotypes that inflicted more damage to the host, independently from their capacity to replicate within the host. The mechanism underlying the reduced tolerance to the infection with the evolved lines deserves further investigation.

There have been few attempts to explore the microevolutionary response of a pathogen facing the conditions provided by a coinfected host. Leggett et al. [40] let a bacteriophage to evolve under a single infection regime or a regime of mixed genotype infections and then assayed the evolved viral lines under the two environments (single or mixed genotype infections). The results showed that viral lines evolved in coinfected bacteria (mixed genotype infections) had a shorter time to lysis (i.e., killed the bacterium faster) compared to the single infection only when assayed under the mixed infection environment. This result is consistent with the idea that when competing with other viral strains, selection promoted time to lysis at the expense of viral yield [40]. Although there are a number of major differences between this work and ours (coinfection involving different genotypes of the same species vs different species, type of interaction between competitors), we believe that the general take home message is that pathogens facing variable environmental conditions in single infected or coinfected hosts can rapidly adjust their strategy of host exploitation as to maximize their fitness.

In line with this argument, an important parameter shaping the fitness consequences following a selection episode is whether organisms face matched or mismatched environmental conditions. For instance, when pathogens are passaged in a novel host, they usually become adapted to this novel host (the matched environment) at the expense of the ancestral host (the mismatched environment) [61]. We found evidence supporting this idea since Py lines passaged in coinfected hosts reached higher parasitemia when assayed in coinfected hosts, compared to the COI-lines assayed in single infected hosts. While in agreement with the theoretical predictions, this result raises the question of the possible epidemiological consequences of the adaptation of *Plasmodium* parasites to helminth-coinfected hosts. In the intertropical zone, there is a large overlap between the areas of endemicity of *Plasmodium* and different species of gastrointestinal nematodes, resulting in high prevalence of coinfection, especially in the most vulnerable populations [16, 35]. *Plasmodium* therefore is likely to consistently experience both the environmental conditions provided by single infected and helminth-coinfected hosts, although other parameters, such as the order of infection, might change between rounds of transmission [21, 34]. When selection fluctuates over time (single infection, coinfection, etc.), plastic responses (as opposed to fixed strategies) are thought to confer the best fitness prospects, possibly maintaining exploitation rules allowing *Plasmodium* to maximize within-host replication and inter-host transmission according to the current environment provided by the host. Deworming campaigns are regularly deployed to protect exposed populations from the debilitating effects of helminths [19, 36, 63]. However, deworming does not eradicate helminths from the entire population and therefore probably contributes to create fluctuating selection on coinfecting microparasites, possibly favoring the plastic adjustments of their host exploitation strategies.

## Conclusion

We found that pre-transmission host mortality reduced the selective benefit of Py lines with the fastest replication rate, possibly resulting in the lack of divergence between Py lines selected in single infected or in coinfected hosts. Helminth-infected hosts are more susceptible to subsequent infection with *Plasmodium* and, in a previous work [21], we showed that Py induces more severe disease symptoms in Hp coinfected hosts. Here, we suggest that pre-transmission host mortality sets a limit to the evolutionary trajectory of virulence in coinfected hosts. We also found evidence for a rapid adaptation to the environmental conditions provided by coinfected hosts, since replication was slowed down during the early phase of the infection when Py passaged in coinfected hosts was tested in single infected hosts. This result might contribute to a better understanding of the epidemiological and public health consequences of deworming campaigns, where *Plasmodium* is likely to experience coinfection conditions prior to the deworming and single infection conditions post deworming. Although extrapolating results obtained under lab conditions on model organisms to the natural conditions is always risky, our results suggest that deworming might prove beneficial both in terms of the direct reduction of the debilitating effect of helminthiasis, and in terms of the indirect reduction of the severity of disease symptoms caused by coinfecting *Plasmodium*.

## Supplementary Information

Additional file 1

## Data Availability

All the data and codes are available in Zenodo (10.5281/zenodo.17953365).
