# Supplementary material for: Experimental evolution of Plasmodium yoelii in single and helminth-coinfected mice
Source: Malar J. 2025 Dec 26;25:63. doi: 10.1186/s12936-025-05764-1 (PMC12849330; doi:10.1186/s12936-025-05764-1)
Supplement: Supplementary file 1 — Additional file 1 [file 12936_2025_5764_MOESM1_ESM.pdf]

Supplementary material

Experimental evolution of *Plasmodium yoelii* in single and helminth-coinfected mice

Aloïs Dusuel[1*], Luc Bourbon[2*], Emma Groetz[1], Mickaël Rialland[1],

Benjamin Roche[3], Bruno Faivre[2], Gabriele Sorci[2#]

1. CTM - Center for Translational and Molecular Medicine, INSERM UMR 1231, Université de Bourgogne, 21000 Dijon, France ; LabEx LipSTIC, 21000 Dijon, France

2. Biogéosciences, CNRS UMR 6282, Université de Bourgogne, 6 Boulevard Gabriel, 21000 Dijon, France

3. MIVEGEC, IRD, CNRS, Université de Montpellier, 34090 Montpellier, France

#corresponding author: gabriele.sorci@u-bourgogne.fr

*equal contribution

Supplementary tables and figures

Table S1. Primer sequences used for the RT-qPCR.

Target	Forward sequence (5’ to 3’)	Reverse sequence (5’ to 3’)
β-Actin	ATGGAGGGGAATACAGCCC	TTCTTTGCAGCTCCTTCGTT
IFN-γ	GAGCTCATTGAATGCTTGGC	GCGTCATTGAATCACACCTG
IL-10	AAGGCTTGGCAACCCAAGTAACCC	TGCACTACCAAAGCCACAAGGCAG

Table S2. Spectral flow cytometry panels.

Emission (nm)	Target	Fluorophore	Clone	Supplier	Cat. ID
Viability					
473	/	LIVE/DEAD Blue	/	Invitrogen	L23105
Surface markers					
388	CD45	BUV395	30-F11	BD Bioscience	564279
615	CD19	BUV615	1D3	BD Bioscience	751213
661	CD8α	BUV661	53-6.7	BD Bioscience	750023
605	CD3	BV605	17A2	BD Bioscience	564009
700	CD4	AF700	RM4-5	BD Bioscience	557956
Intracellular markers					
774	FOXP3	PE-Cy7	FJK-16S	Invitrogen	25-5773-82

Table S3. Model selection procedure. For each response variable, we started with the full model (first line), we simplified it and we compared the AICc values. The model highlighted in green corresponds to the lowest AICc value. Equivalent models (∆ AICc < 2) are highlighted in orange and were averaged (see below).

Response variable	Models	AICc	∆ AICc value	Explanatory variable code
Log-ratios of body mass (see table 1 in the main text of the article)	A+B+C+A*B+A*C+B*C+A*B*C	-815.5	78.3	A = Treatment
	A+B+C+A*B+A*C+B*C	-864.5	29.3	B = Passage
	A+B+C+A*B+A*C	-874.6	19.2	C = Time p.i.
	A+B+C+A*B+B*C	-878.1	15.7	
	A+B+C+A*C+B*C	-860.6	33.2	
	A+B+C+A*B	-888.5	5.3	
	A+B+C+A*C	-870.3	23.5	
	A+B+C+B*C	-873.8	20	
	A+B+C	-883.5	10.3	
	A+B	-726.5	167.3	
	A+C	-887.3	6.5	
	B+C	-890	3.8	
	A	-728.3	165.5	
	B	-733	160.8	
	C	-893.8	0	
	Intercept-only	-735.2	158.6	
Log-ratios of RBC (see table 2 in the main text of the article)	A+B+C+A*B+A*C+B*C+A*B*C	-95.4	12.8	A = Treatment
	A+B+C+A*B+A*C+B*C	-98.7	9.5	B = Passage
	A+B+C+A*B+A*C	-102.2	6	C = Time p.i.
	A+B+C+A*B+B*C	-104.5	3.7	
	A+B+C+A*C+B*C	-98.4	9.8	
	A+B+C+A*B	-108.1	0.1	
	A+B+C+A*C	-102.8	5.4	
	A+B+C+B*C	-103.6	4.6	
	A+B+C	-107.9	0.3	
	A+B	66.3	174.5	
	A+C	-108.2	0	
	B+C	-105	3.2	
	A	63.2	171.4	
	B	71.5	179.7	
	C	-105.5	2.7	
	Intercept-only	68.8	177	
Parasitemia during passages (see table 3 in the main text of the article)	A+B+A*B	-118.78	0	A = Treatment
	A+B	-114.22	4.56	B = Passage
	A	-103.95	14.83	
	B	-114.19	4.59	
	Intercept-only	-104.22	14.56	
Logit-ratio of parasitemia (see table 4 in the main	A+B+C+A*B+A*C+B*C+A*B*C	561.9	0	A = Treatment
	A+B+C+A*B+A*C+B*C	579.6	17.7	B = Passage
	A+B+C+A*B+A*C	599.6	37.7	C = Time p.i.
	A+B+C+A*B+B*C	588.2	26.3	

text of the article)	A+B+C+A*C+B*C<br>A+B+C+A*B<br>A+B+C+A*C<br>A+B+C+B*C<br>A+B+C<br>A+B<br>A+C<br>B+C<br>A<br>B<br>C<br>Intercept-only	578.5<br>602.6<br>598.4<br>586.9<br>601.5<br>662.1<br>600.2<br>599.6<br>660.2<br>660.2<br>598.3<br>658.3	16.6<br>40.7<br>36.5<br>25<br>39.6<br>100.2<br>38.3<br>37.7<br>98.3<br>98.3<br>36.4<br>96.4	
Log-ratio of EPO (see text of the article)	A<br>Intercept-only	65<br>66.6	0<br>1.6	A = Treatment
Log-ratio of HO-1 (see text of the article)	A+B+A*B<br>A+B<br>A<br>B<br>Intercept-only	27.3<br>26.1<br>24.6<br>30.2<br>28.7	2.7<br>1.5<br>0<br>5.6<br>4.1	A = Treatment<br>B = Passage
Log-log regression between RBC counts and parasitemia (see table 5 in the main text of the article)	A+B+A*B<br>A+B<br>A<br>B<br>Intercept-only	141.2<br>150.1<br>447.1<br>148.2<br>447.2	0<br>8.9<br>305.9<br>7<br>306	A = Treatment<br>B = Parasitemia
ΔΔ Ct IFN-γ (see table 6 in the main text of the article)	A+B+A*B<br>A+B<br>A<br>B<br>Intercept-only	105.6<br>116.9<br>125<br>113.6<br>124.2	0<br>11.3<br>19.4<br>8<br>18.6	A = Treatment<br>B = Time p.i.
ΔΔ Ct IL-10 (see table 7 in the main text of the article)	A+B+A*B<br>A+B<br>A<br>B<br>Intercept-only	125<br>128.2<br>130<br>132.4<br>133.9	0<br>3.2<br>5<br>7.4<br>8.9	A = Treatment<br>B = Time p.i.
Logit-ratio of Treg (see table 8 in the main text of the article)	A+B+A*B<br>A+B<br>A<br>B<br>Intercept-only	-42.9<br>-46.1<br>-41.4<br>-41.2<br>-34.5	3.2<br>0<br>4.7<br>4.9<br>11.6	A = Treatment<br>B = Passage

Parasitemia of COI-lines in matched or mismatched environments (see table 9 in the main text of the article)	A+B+A*B<br>A+B<br>A<br>B<br>int	298.43<br>323.5<br>468.61<br>331.81<br>466.38	0<br>25.07<br>170.18<br>33.38<br>167.95	A = Environment<br>B = Passage

Table S4. Parameter estimates with the 95% confidence intervals for the averaged model exploring the log-ratios of RBC counts.

**Fixed effects**			**Estimate**	**95% CI**
Treatment				
	COI-lines		0.076	0.020/0.132
Passage				
		7	-0.014	-0.065/0.038
Time p.i.				
	3		-0.013	-0.053/0.026
	9		-0.572	-0.666/-0.477
	14		-0.414	-0.477/-0.351
Treatment x Passage				
	COI-lines	7	-0.027	-0.098/0.045

Table S5. Parameter estimates with the 95% confidence intervals for the averaged model exploring the log-ratios of HO-1.

**Fixed effects**			**Estimate**	**95% CI**
Treatment				
	COI-lines		-0.336	-0.553/-0.120
Passage				
		7	-0.037	-0.187/0.110

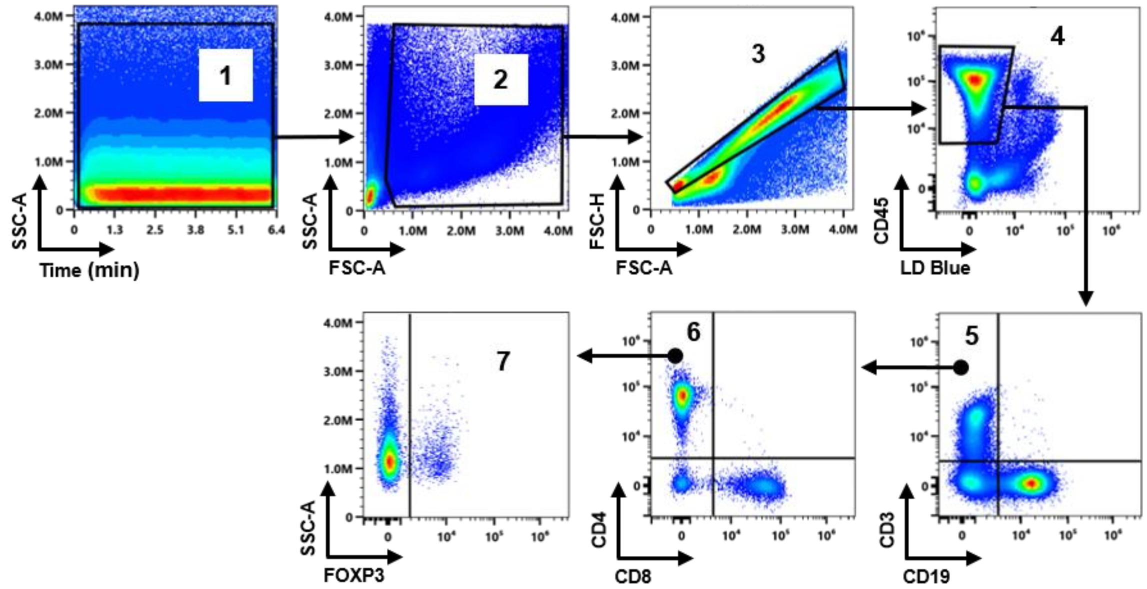

Figure S1. Gating strategy used in flow cytometry analysis to identify Treg cell subset within mouse total splenocytes. (1) Time parameter to exclude microfluidic fluctuations. Selection of (2) splenocytes without debris and (3) single cells. Selection of (4) live LIVE/DEAD-Blue$^-$ and immune CD45$^+$ cells. Selection of (5) CD19$^-$CD3$^+$ T cells. Selection of (6) CD8$^-$CD4$^+$ helper T cells. Selection of (7) FOXP3$^+$ Treg cell subset within CD4$^+$ T lymphocytes.

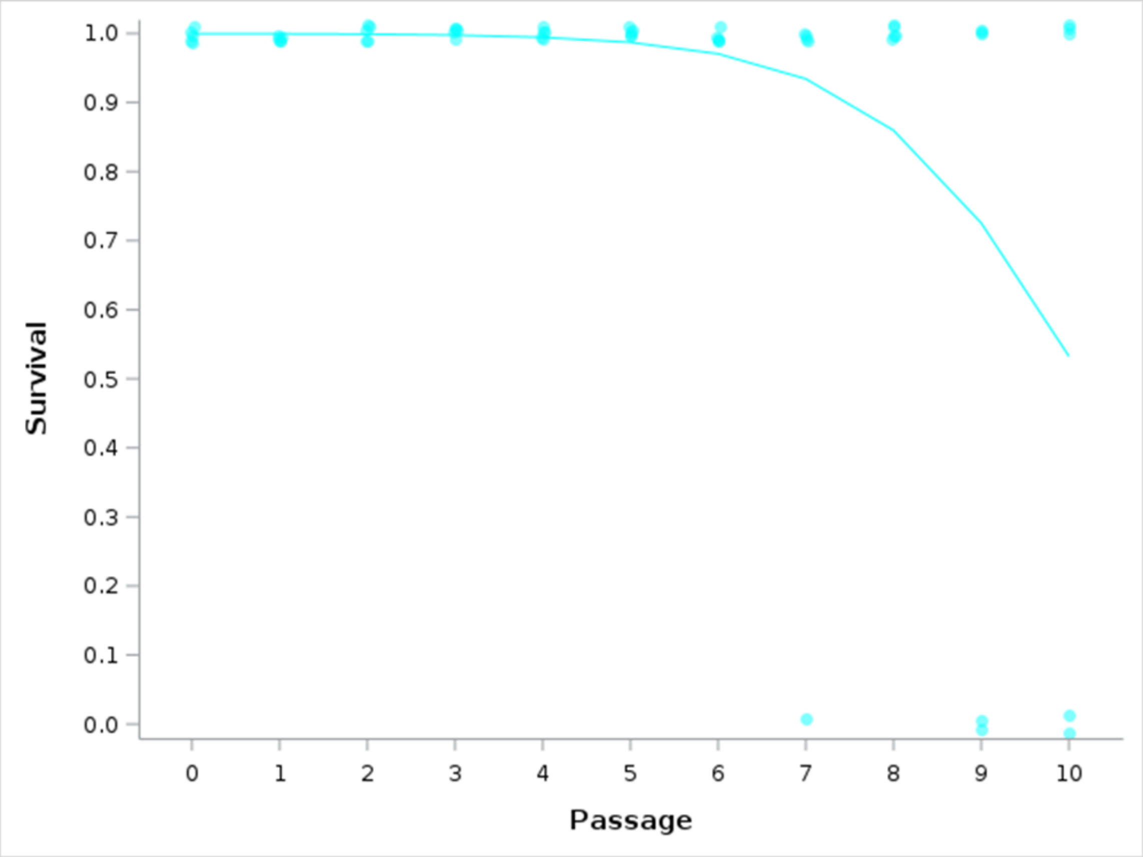

Figure S2. Proportion of single infected hosts alive at day 14 p.i. over the passages. The dots represent the raw data (1 = alive, 0 = dead) and the line represents the fit of a GLM with a binomial distribution or errors. N = 5 hosts per passage.

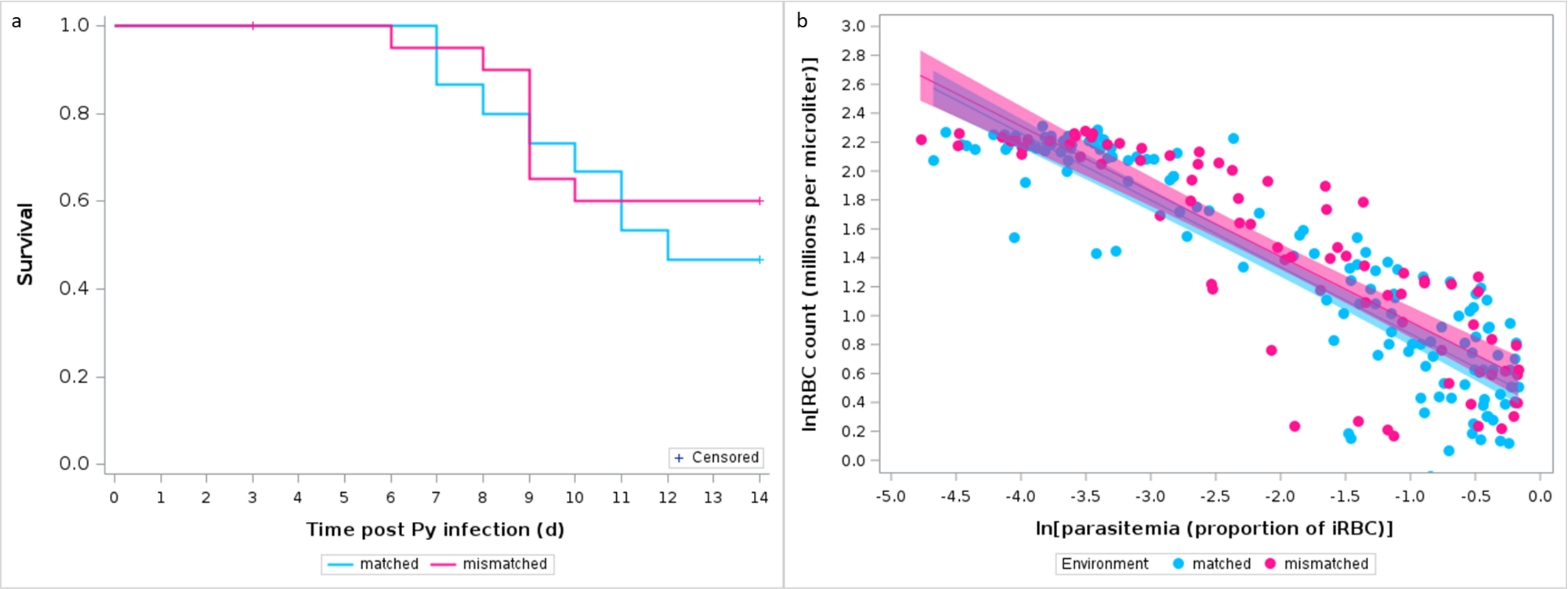

Figure S3. a) Survival up to day 14 p.i. of hosts infected with COI-lines after 7 passages that experienced matched environments between the passages and the evaluation trials (coinfection/coinfection, n = 25) or mismatched environments (coinfection/single infection, n = 30). Crosses indicate censored observations. b) Log-log plot of the relationship between RBC count and parasitemia in hosts infected with COI-lines after 7 passages that experienced matched environments between the passages and the evaluation trials (coinfection/coinfection) or mismatched environments (coinfection/single infection). The dots represent the raw data, the line and the shaded area the LMM fit with the 95% CI.
